# Supplementary material for: Understanding networks in low-and middle-income countries’ health systems: A scoping review
Source: PLOS Glob Public Health. 2023 Jan 11;3(1):e0001387. doi: 10.1371/journal.pgph.0001387 (PMC10022031; doi:10.1371/journal.pgph.0001387)
Supplement: S1 Table — (DOCX) [file pgph.0001387.s001.docx]

| **Inclusion** | **Exclusion** |
| --- | --- |
| - Related to health system networks - Networks: groups of facilities and/or healthcare stakeholders (including but not limited to all types of providers, technicians, government officials, professional associations, NGOs, and donors) linked formally or informally, horizontally or vertically, through programs, interventions, activities, or initiatives - Health systems: structures, processes, and people responsible for managing health programs and services that provide care for a population - HICs and LMICs | - The program, intervention, activity, or initiative occurs only in one facility or locality among only one group of actors - Research or purely academic networks - Networks focused on research capacity building - Communities of practice - Advocacy networks - Disaster management networks - Database/registry networks - Trial/study networks - Social networks - Family/home care networks - Palliative care networks - Laboratory networks - Diagnostics networks - Disease surveillance networks - Telemedicine/e-health/m-health interventions - Accreditation interventions - Integrated community case management interventions - Integrated management of childhood illnesses interventions - Mobile unit outreach - Faith based organization sector - General studies on Universal Health Care (UHC) - Primary Health Care networks that were limited to the peripheral level of the health system |

S1 Table. Literature eligibility criteria
