# Supplementary material for: Understanding networks in low-and middle-income countries’ health systems: A scoping review
Source: PLOS Glob Public Health. 2023 Jan 11;3(1):e0001387. doi: 10.1371/journal.pgph.0001387 (PMC10022031; doi:10.1371/journal.pgph.0001387)
Supplement: S2 Table — (DOCX) [file pgph.0001387.s002.docx]

| Study  # | Citation | Intervention Country | Aim/  Purpose of Study | Study Methodology | Intervention | Study Outcomes/ Main Findings | Network  Stakeholders | Type of Network | Network Definition | Network Characteristics | Network Use | Network Purpose | Other key findings |
| --- | --- | --- | --- | --- | --- | --- | --- | --- | --- | --- | --- | --- | --- |
| Study 1 |  |  |  |  |  |  |  |  |  |  |  |  |  |
| Study 2 |  |  |  |  |  |  |  |  |  |  |  |  |  |
| Study…. |  |  |  |  |  |  |  |  |  |  |  |  |  |

S2 Table. Data charting instrument
