# Supplementary material for: Understanding networks in low-and middle-income countries’ health systems: A scoping review
Source: PLOS Glob Public Health. 2023 Jan 11;3(1):e0001387. doi: 10.1371/journal.pgph.0001387 (PMC10022031; doi:10.1371/journal.pgph.0001387)
Supplement: S3 Table — (DOCX) [file pgph.0001387.s003.docx]

| **Author** | **Type of network** | **Typology / Framework** | **HIC / LMIC** | **Framework elements*** |
| --- | --- | --- | --- | --- |
| Ferlie et al., 2010 | Managed networks  Research / academic networks | Typology | HIC | - Complexity of context - **Network form** - **Resource base** - **Formalization** - Range of stakeholders - **Processes and skills** |
| Brown, Patel et al., 2016 | Clinical Networks | Typology | HIC | - Definition - Membership - **Governance and management** |
| Sheaff and Schofield, 2016 | Inter-organizational networks | Characteristics | HIC | - Environment - **Structure** - **Process** - Outcomes |
| Provan and Milward, 2001 | Public sector organizational networks | Network effectiveness frameworks | HIC | - Effectiveness criteria |
| Turrini, 2010 | Public networks | Network effectiveness frameworks | HIC | - **Structural** - **Functioning** - Contextual |
| Brown, Haines et al., 2016 | Clinical network | Domains to measure features of clinical networks | HIC | - Engagement of multidisciplinary clinicians - **Leadership** - **Strategic and operational management** - External support - Network value |
| Gunst, 2016 | n/a | Clinical governance framework | LMIC | - **Governance** - Services - **Critical support functions** - Quality |

S3 Table. Existing network frameworks and typologies identified

Bold phases contributed to the development of the proposed framework. Context, environment, and outcomes were not included in the proposed framework as they will be investigated in depth in a subsequent realist review.
