# Supplementary material for: Understanding networks in low-and middle-income countries’ health systems: A scoping review
Source: PLOS Glob Public Health. 2023 Jan 11;3(1):e0001387. doi: 10.1371/journal.pgph.0001387 (PMC10022031; doi:10.1371/journal.pgph.0001387)
Supplement: S4 Table — (DOCX) [file pgph.0001387.s004.docx]

| **Component** | **Most frequent practical characteristic** | **# of references** | **Example from the literature** | **Example citation** |
| --- | --- | --- | --- | --- |
| **Form and structure** | Connecting across levels of the health system and entities | 41 | - Network actors mapped referral pathways to link public and private referral hospitals and community health centers and providers - Set connections between different levels of staff and health system managers | Hyre A et al. Expanding Maternal and Neonatal Survival in Indonesia: A program overview. International Journal of Gynecology & Obstetrics. 2019; 144. doi: 10.1002/ijgo.12730. |
|  | Established vision, mission, shared values, targets, rules, roles, responsibilities, culture | 35 | - Social franchise network offered services under a common brand with a specific social mission | Syengo M et al. Private Providers' Experiences Implementing a Package of Interventions to Improve Quality of Care in Kenya: Findings from a Qualitative Evaluation. Global Health-Science and Practice. 2020; 8:3, 478-487. doi: 10.9745/GHSP-D-20-00034. |
|  | Partnerships and links to external stakeholders | 29 | - Created partnerships and linkages with the regional diabetes associations, MoH, specialists, partner organizations | Diallo MM et al. Access to diabetes care in sub-Saharan Africa: Results of a diabetes health network in Guinea. Medecine des Maladies Metaboliques. 2013; 7:3, 272-276. |
|  | Network agreements and mapping | 29 | - Established a MoU with MoHCDGEC, a LoA with RMO, and a LoAs with sites where providers are placed to establish and govern functioning of network | D'Mello BS et al. Averting Maternal Death and Disability in an Urban Network of Care in Dar es Salaam, Tanzania: A Descriptive Case Study. Health Systems & Reform. 2020; 6:2. e1834303. doi: 10.1080/23288604.2020.1834303. |
|  | Linkages, engagement, and alignment with government | 30 | - Created strong relationships with the MoH, which was solidified through MoUs - Held annual joint planning with district - Developed informal relationships with civil authorities - Performed joint supervision | Cordier LF et al. Networks of Care in Rural Madagascar for Achieving Universal Health Coverage in Ifanadiana District. Health Systems & Reform. 2020; 6:2, e1841437. doi: 10.1080/23288604.2020.1841437. |
| **Governance and leadership** | Network meetings | 36 | - Held monthly meetings between TBAs, CHWs, and health center staff - Held monthly quality improvement team meeting | Broughton E et al. Evaluation of an intervention to improve essential obstetric and newborn care access and quality in Cotopaxi, Ecuador. Frontiers in Public Health. 2016; 4:247. doi: 10.3389/fpubh.2016.00247. |
|  | Network leadership and management | 31 | - Local network leadership ensured success of the network | Nahimana E et al. Race to the Top: evaluation of a novel performance-based financing initiative to promote healthcare delivery in rural Rwanda. Global Health Action. 2016; 9:1, 32943, doi: 10.3402/gha.v9.32943. |
|  | Working groups and taskforces | 23 | - Developed national task forces for strategic insight, coordination, and oversight - Established technical expert groups to support decision- making and implementation | Gudlavalleti VSM et al. Public health system integration of avoidable blindness screening and management, India. Bulletin of the World Health Organization. 2018; 96:10, 705-715. doi: 10.2471/BLT.18.212167. |
|  | Government leadership and oversight | 21 | - Apex CHOs supervised TBAs (linked to PHCB) - Local Government Authority held data review meetings to monitor care within the network | Fasawe O et al. Applying a Client-centered Approach to Maternal and Neonatal Networks of Care: Case Studies from Urban and Rural Nigeria. Health Systems & Reform. 2020; 6:2, e1841450. doi: 10.1080/23288604.2020.1841450. |
|  | Network manager, coordinator, or facilitator | 18 | - Clinical coordinators managed interdisciplinary QI team - Regional coordinators supported clinics - National coordinators | Chan BTB et al. A programme to improve quality of care for patients with chronic diseases, Kazakhstan. Bulletin of the World Health Organization. 2020; 98:3, 161-169. doi:10.2471/BLT.18.227447. |
| **Functioning** | Knowledge and information sharing, education, and learning | 65 | - Quality improvement collaborative learning sessions held to share progress, ideas, best practices, lessons learned | Dougherty G et al. Reaching the First 90: Improving Inpatient Pediatric Provider-Initiated HIV Testing and Counseling Using a Quality Improvement Collaborative Strategy in Tanzania. Journal of the Association of Nurses in AIDS Care. 2019; 30:6, 682-690. doi:10.1097/JNC.0000000000000066. |
|  | Guideline, standards, and protocols uptake and adherence | 56 | - Data shared for the implementation and evaluation of strategies to improve guideline uptake | Akech S et al. Magnitude and pattern of improvement in processes of care for hospitalised children with diarrhoea and dehydration in Kenyan hospitals participating in a clinical network. Tropical Medicine and International Health. 2019; 24:1, 73-80. |
|  | Data collection, analysis, use, and quality | 56 | - Revived and expanded community-based health management information system to report births and deaths - LGA data review meetings to report trends and gaps to solve data quality issues, identify implementation issues, and share successes | Sloan NL et al. Advancing Survival in Nigeria: A Pre-post Evaluation of an Integrated Maternal and Neonatal Health Program. Maternal and Child Health Journal. 2018; 22:986-997. Doi: 10.1007/s10995-018-2476-3. |
|  | Quality improvement | 50 | - Quality improvement collaborative improved person-centered care for maternal health and family planning services | Giessler K et al. Perspectives on implementing a quality improvement collaborative to improve person-centered care for maternal and reproductive health in Kenya. International Journal for Quality in Health Care. 2020; 30:10, 671-676. doi:10.1093/intqhc/mzaa130. |
|  | Care pathways and models of service delivery promotion and implementation | 46 | - Implemented referral network with ambulance transport | Tayler-Smith K et al. An ambulance referral network improves access to emergency obstetric and neonatal care in a district of rural Burundi with high maternal mortality. Tropical Medicine & International Health. 2013; 18:8, 993-1001. doi:10.1111/tmi.12121. |
| **Resources** | Human resources | 43 | - Mid-level managers act as the network’s focal points and play “boundary spanning roles” from clinical to management | Irimu G et al. Approaching quality improvement at scale: a learning health system approach in Kenya. Arch Dis Child. 2018. doi: 10.1136/archdischild-2017-314348. |
|  | IT | 38 | - mhealth platform used for perinatal monitoring and referral by TBAs | Martinez B et al. mHealth intervention to improve the continuum of maternal and perinatal care in rural Guatemala: a pragmatic, randomized controlled feasibility trial. Reproductive Health. 2018; 15:120. doi: 10.1186/s12978-018-0554-z. |
|  | Commodities and equipment | 14 | - Network provided infrastructure, equipment, and supply chain support | Cordier LF et al. Networks of Care in Rural Madagascar for Achieving Universal Health Coverage in Ifanadiana District. Health Systems & Reform. 2020; 6:2, e1841437. doi: 10.1080/23288604.2020.1841437. |
|  | Funding | 13 | - Business support interventions enabled access to the Medical Credit Fund, linking providers to banks for low interest rate loans to procure equipment and upgrade facilities | Syengo M et al. Private Providers' Experiences Implementing a Package of Interventions to Improve Quality of Care in Kenya: Findings from a Qualitative Evaluation. Global Health-Science and Practice. 2020; 8:3, 478-487. doi: 10.9745/GHSP-D-20-00034. |
|  | Government funding | 12 | - Municipality funds used to pay contracted providers and invest in health activities, including the continuation and expansion of network initiatives | Bhatta S et al. The Logarithmic Spiral of Networks of Care for Expectant Families in Rural Nepal: A Descriptive Case Study. Health Systems and Reform. 2020; 6:2. doi:10.1080/23288604.2020.1824520. |
|  | Supportive policies | 12 | - Existing policies encouraged facility-based births and provision of high-quality maternal and neonatal care | Hyre A et al. Expanding Maternal and Neonatal Survival in Indonesia: A program overview. International Journal of Gynecology & Obstetrics. 2019; 144. doi: 10.1002/ijgo.12730. |
| **Communication** | Communication between network members | 22 | - Communication between the network members helped to facilitate trusting relationships between network members of different cadres, sectors, and levels of care | Vergara MT et al. Building Trust to Save Lives in a Metro Manila Public-Private Network of Care: A Descriptive Case Study of Quirino Recognized Partners in Quezon City, Philippines. Health Systems & Reform. 2020; 6:2, e1815473, doi: 10.1080/23288604.2020.1815473. |
|  | Communication infrastructure | 11 | - 24h communications network was implemented to facilitate referral | Tayler-Smith K et al. An ambulance referral network improves access to emergency obstetric and neonatal care in a district of rural Burundi with high maternal mortality. Tropical Medicine & International Health. 2013; 18:8, 993-1001. doi:10.1111/tmi.12121. |
|  | Strengthening communication | 5 | - The audit of a referral network strengthened communication for referrals | Awoonor-Williams JK et al. Conducting an audit to improve the facilitation of emergency maternal and newborn referral in northern Ghana. Global Public Health. 2015; 10:9, 1118-1133. doi: 10.1080/17441692.2015.1027247. |
|  | Effective communication strategies | 4 | - Regular meetings were held between different cadres of providers | Mullany LC et al. The MOM Project: Delivering maternal health services among internally displaced populations in eastern Burma. Reproductive Health Matters. 2008; 16:31, 44-56. doi: 10.1016/S0968-8080(08)31341-X. |

S4 Table. Most frequent practical characteristics by network component with examples from the selected literature
