# Supplementary material for: Understanding networks in low-and middle-income countries’ health systems: A scoping review
Source: PLOS Glob Public Health. 2023 Jan 11;3(1):e0001387. doi: 10.1371/journal.pgph.0001387 (PMC10022031; doi:10.1371/journal.pgph.0001387)
Supplement: S2 Appendix — (DOCX) [file pgph.0001387.s006.docx]

## S6 Appendix. Characteristics of Sources of Evidence

| **#** | **Citation** | **Intervention Country** | **Aim/purpose of study** | **Study Methods** | **Intervention** | **Outcomes/Main Findings** | **Type of Network** |
| --- | --- | --- | --- | --- | --- | --- | --- |
| **Peer-review literature** | | | | | | | |
| 1 | Abdul Kader MAS. Strengthening acute coronary syndrome referral network: Insights from initiatives of Penang General Hospital cardiology centre. Medical Journal of Malaysia. 2019; 74: 4, 355-358. | Malaysia | Commentary on proof of concept to share experiences and give an overview of the initiative | Audit and intervention based on areas needed for improvement | Development and implementation of standardized protocols in all referral hospitals, referral network workshop as medical continuing education for medical officers in referral hospitals | Network resulted in improved quality of PCI referrals | -State PCI-referral network with large tertiary hospital at center -Hub-spoke model |
| 2 | Abul-Fadl A, Abu-Zeid A, Besher M, and Sarhan AE. Assessing needs for use of communication networks for continuous improvement of primary health care services. International Proceedings of Chemical, Biological and Environmental Engineering. 2012; 41:87-92. | Egypt | To identify the acceptability, accessibility, affordability, and efficiency for IT for telehealthcare networks in PHC | -Pilot study assessed by interviews to understand access, skills, and practice use -Assessed needs for teaching primary care medicine using technology and telecommunications by networking universities with PHCs to reinforce teaching in PHC settings | IT solution installed in three large universities in three regions and linked with six remote PHCs | Telecommunications can improve service delivery at PHCs and is a cost-effective way to improve outcomes | Telehealth network: linked university and PHCs centres for teaching, training, and consultation |
| 3 | Adams V, Craig S, Samen A, and Bhatta S. It Takes More than a Village: Building a Network of Safety in Nepal's Mountain Communities. Maternal and Child Health Journal. 2016; 20:19, 2424-2430. | Nepal | To show how an NGO established a model for integrated healthcare delivery and support | Report from the field | Implementation of networks of safety in mountainous regions of Nepal, including:  -Education for family and community (FCHV, CHWs) -Provider trainings -Collaboration with district-level partners on MCH strategic plans and MoH with national policies -Addresses infrastructure gaps (facility and referral hospitals) and obstacles to safe delivery through investment in improvement of facilities | Decrease in maternal deaths | Network of safety - from families to villages up to tertiary care and MoH |
| 4 | Addicott R and Ferlie E. Understanding power relationships in health care networks. Journal of health organization and management. 2007; 21: 4/5, 393-405. DOI 10.1108/14777260710778925 | UK (London) | To show that networks are becoming a new, innovative organizational form in the UK public sector and explore and theorize the nature of power relations within a network model of governance | -Comparative case studies -Semi structured interviews, document analysis, meeting observation -Review of evidence of 5 case studies on cancer managed care networks in London | Cancer managed care networks and power relations within the network | "A model of bounded pluralism can be used to understand power relations within London MCNs. However, power over the development of policy and strategic direction is instead exerted in a top-down manner by the government (e.g. Department of Health) and its associated national bodies" | Managed care network |
| 5 | Akech S, Ayieko P, Irimu G, Stepniewska K, and English M. Magnitude and pattern of improvement in processes of care for hospitalised children with diarrhoea and dehydration in Kenyan hospitals participating in a clinical network. Tropical Medicine and International Health. 2019; 24:1, 73-80. | Kenya | To describe the magnitude and patterns of changes in processes of care for children with diarrhea and dehydration in a clinical network | -Observational study -Pediatric Admission Quality of Care scoring | -Implementation of CIN: data on admissions, clinical information, treatment and discharge information entered into an online database by trained clerk after child discharge -Data synched to a central server -Reports produced on processes of care every 2-3 months and shared with the hospitals with a combined report on performance of all hospitals, enabling comparison of hospital performance -Reports are disseminated and discussed by Pediatrician and clinical team to identify action plans -Pediatricians are mentored on how to give feedback and develop action plans -Follow-up from KWTRP after reports to discuss improvement or deterioration -Twice yearly CIN meetings to share experiences with key personnel | -"Adherence to guidelines for children admitted with diarrheal disease can be improved through participation in a clinical network but improvement is limited, not uniform for all aspects of care and contexts and occurs early" -Mean PAQC score increased by 13.8% in the first 12 months, average 0.9% increase/month but then plateaued -Changes were similar in the two groups of hospitals joining the network at different times | Clinical Information Network |
| 6 | Alderman EM, Freeman KL, and Lobach KS. Improving adolescent access and services in a large primary care network: report of a 10 year project. International Journal of Adolescent Medicine and Health. 2019; 31:2. | USA | Narrative report: overview of MAPCI initiative in the MMG network | -Baseline survey and provider conference -Subsequent studies on different aspects of the network | -Needs assessment (baseline survey and provider summit) to evaluate current state of adolescent PHC services within the network and develop recommendations -Development an initiative to create a uniform approach to adolescent primary care in the network -Steering committee and working groups to implement initiative | Improvement of adolescent health services in a large PHC network | -Primary care network: community-based practices and school health clinics linked to a large academic medical center -6/22 sites are resident training sites -Some sites are linked to another community health network as well |
| 7 | Alkmim MB, Figueira RM, Marcolino MS, Cardoso CS, de Abreu MP, Cunha LR, da Cunha DF, Antunes A P, Resende AGD, Resende ES, and Ribeiro ALP. Improving patient access to specialized health care: the Telehealth Network of Minas Gerais, Brazil. Bulletin of the World Health Organization. 2012; 90: 373-378. doi:10.2471/BLT.11.099408. | Brazil | Determine user satisfaction and cost-effectiveness of tele-health network | -Project overview -User satisfaction surveys -Cost-effectiveness analyses | -Establish telehealth network linking university hospitals with remote municipal health departments -Provide support using tele-assistance, conduct tele-electrocardiography and teleconsultations for a variety of issues | "a telehealth service must be part of a collaborative network, meet the real needs of local health professionals, use simple technology and have at least some face-to-face components. If applied to health problems for which care is in high demand, this type of service can be economically viable and can help to improve patient access to specialized health care" | Telehealth network: university hospitals linked to remote municipal health departments in the state, connects specialists with PHC providers |
| 8 | Amiel JM and Pincus HA. The medical home model: new opportunities for psychiatric services in the United States. Current Opinion in Psychiatry. 2011; 24:6, 562-8. doi:10.1097/YCO.0b013e32834baa97. | USA | Overview of PCMH model and how it was adopted for the delivery of psychiatric care | Review | Integration of psychiatric care into PCMH | "PCMH model offers a useful conceptual framework for the management of complex and chronic psychiatric illness. Early pilots of its use in psychiatric settings have demonstrated that people with psychiatric illness who receive their care in a medical home have better access to care, improved response to treatment, and higher cost efficiency compared with usual care" | Patient centered medical home |
| 9 | Arrieta J, Orrego C, Macchiavello D, Mora N, Delgado P, Giuffre C, Elorrio EG, Rodriguez V. 'Adios Bacteriemias': a multi-country quality improvement collaborative project to reduce the incidence of CLABSI in Latin American ICUs. International Journal for Quality in Health Care. 2019; 31:9,704-711. doi: 10.1093/intqhc/mzz051. | Five countries in Latin America: Argentina, Chile, Colombia, Mexico, Uruguay | Evaluation | -Explanation of collaborative  -Descriptive statistics on an uncontrolled before-after comparison of CLABSI rates | Quality improvement collaborative focused on the implementation of bundles of care for insertion and maintenance of central lines | -Overall reduction in the CLABSI incidence rate of 22% during the intervention period -Quality improvement collaborative effective in reducing the incidence of CLABSI and improving adherence to good practices and maintenance processes in participating ICUs | Multi-country quality improvement collaborative |
| 10 | Aspromonte N, Gulizia MM, Di Lenarda A, Mortara A, Battistoni I, De Maria R, Gabriele M, Iacoviello M, Navazio A, Pini D, Di Tano G, Marini M, Ricci RP, Alunni G, Radini D, Metra M, and Romeo F. ANMCO/SIC Consensus Document: Cardiology networks for outpatient heart failure care. European Heart Journal. 2017; 19 Supplement D; D89-D101. doi:10.1093/eurheartj/sux009. | Italy | Guidance document for the organization of national heart failure care network: tasks and requirements of health system points of contact and to define how diagnosis, management, and care processes should be documented and shared | Guidance document | Different levels of heart failure clinics integrated into a dedicated network for management of patients on a regional basis, according to geography | Guidance document with roles and interactions of cardiology specialists | National network for heart failure patients |
| 11 | Austin A, Gulema H, Belizean M, Colaci DS, Kendall T, Tebeka M, Hailemariam M, Bekele D, Tadesse L, Berhane Y, and Langer A. Barriers to providing quality emergency obstetric care in Addis Ababa, Ethiopia: Healthcare providers' perspectives on training, referrals and supervision, a mixed methods study. BMC Pregnancy and Childbirth. 2015; 15:74. doi: 10.1186/s12884-015-0493-4. | Ethiopia | Assess barriers to provision of emergency obstetric care in Addis according to providers, focusing on implementation of national referral guidelines, staff training, staff supervision | Mixed methods: semi-structured interviews, key informant interviews, quantitative survey | Establishment of obstetric referral network | "lack of transportation and communication infrastructure, overcrowding at the referral hospital, insufficient pre-service and in-service training, and absence of supportive supervision as key barriers to provision of quality emergency obstetric care" | Urban regional emergency obstetrics referral network |
| 12 | Aveling EL, Martin GP, Armstrong N, Banerjee J, and Dixon-Woods M. Quality improvement through clinical communities: eight lessons for practice. Journal of Health Organization and Management. 2012; 26:2. | Non-specific | Examine role of network forms of organization in QI and to draw out practical lessons to achieve change through this approach | -"author-based" approach review -Used principles of critical interpretive synthesis | Clinical communities for QI | "A number of overlapping but distinct clinical community-based approaches can be identified in the literature, each suitable for different problems. The evidence for the effectiveness of these is mixed, but there is some agreement on the challenges that those adopting such approaches need to address, and how these can be surmounted" | Clinical community for QI |
| 13 | Awoonor-Williams, JK, Bailey P, Yeji F, Adongo AE, Baffoe P, Williams A, and Mercer S. Conducting an audit to improve the facilitation of emergency maternal and newborn referral in northern Ghana. Global Public Health. 2015; 10:9, 1118-1133. doi: 10.1080/17441692.2015.1027247. | Ghana | Audit of referral networks to identify areas for improvement and solve issues in a way that could be modelled elsewhere | Clinical/criteria-based audit of obstetric and newborn referrals in five referral networks | Linking communities with sub-district, district, and regional-level health facilities and improving referral practices between facilities through audit which encouraged positive change in facilitation of emergency referrals | -Increase facilitating referral mechanisms -Decrease in reliance on taxis and increase in use of national/facility ambulance/vehicle -Increase in healthcare worker accompanying referrals -Increase in use of referral slips -Increase in notification to facility where patient is being referred -Increased feedback | Referral networks |
| 14 | Ayieko P, Irimu G, Ogero M, Mwaniki P, Malla L, Julius T, Chepkirui M, Mbevi G, Oliwa J, Agweyu A, Akech S, Were F, English M, Tuti T, Gathara D, Gachau S, Ngarngar S, Injira I, Kimutai D, Emadau C, Mutiso C, Nzioki C, Mithamo A, Kuria M, Otido S, Wachira G, Njiiri P, Inginia R, Musabi M, Charo S, Ochieng G, Thuranira L, and Clinical Information Network, Authors. Effect of enhancing audit and feedback on uptake of childhood pneumonia treatment policy in hospitals that are part of a clinical network: a cluster randomized trial. Implementation Science. 2019; 14:20. doi:10.1186/s13012-019-0868-4. | Kenya | To examine if providing enhanced audit and feedback compared to routine feedback could accelerate uptake of new pneumonia guidelines by clinical teams | Parallel group cluster randomized controlled trial | -Standard intervention: training on new guidelines, dissemination of treatment guidelines, support to improve collection and quality of patient data, mentorship, team management training, peer-to-peer networking, feedback reports on guideline uptake  -Enhanced: in addition to the standard intervention, monthly hospital-specific feedback sheets on pneumonia indicators linked to performance goals, action plans, and follow-up email from network coordinator | -Proportion of children correctly classified and diagnosed in enhanced and standard feedback groups were similar -Difference in adoption of pneumonia policy overtime in the enhanced feedback group vs the standard feedback group | Clinical Information Network |
| 15 | Bailie J, Cunningham FC, Bainbridge RG, Passey ME, Laycock AF, Bailie RS, Larkins SL, Brands JSM, Shanthi Ramanathan, Abimbola S, and Peiris D. Comparing and contrasting 'innovation platforms' with other forms of professional networks for strengthening primary healthcare systems for Indigenous Australians. BMJ Global Health. 2018; 3:3:e000683. doi:10.1136/bmjgh-2017-000683. | Australia | Compare and contrast concept of innovation platforms with other networks to strengthen PHC | Review | Applying an innovation platform approach to integrated quality improvement in Indigenous PHC | "Policymakers should support further experimentation with innovation platforms as a strategy for engaging with multiple stakeholders in health systems strengthening initiatives" | Innovation platform |
| 16 | Bansal N. Burstein DS, Lorts A, Smyth L, Rosenthal DN, and Peng DM. Heart failure in children: Priorities and approach of the ACTION collaborative. Progress in Paediatric Cardiology. 2020; 59. doi: 10.1016/j.ppedcard.2020.101313. | International | Describe the ACTION network | Overview of network | Development of the Advanced Cardiac Therapies Improving Outcomes Network | Decrease in stroke rates of children with VADs across the network | Learning health system/learning network |
| 17 | Bari S, Mannan I, Rahman MA, Darmstadt GL, Seraji MHR, Baqui AH, El Arifeen S, Rahman SM, Saha SK, Ahmed A, Ahmed, S, Santosham M, Black RE, Winch P J, and Bangladesh Projahnmo II. Trends in use of referral care of sick newborns in hospital services for a community-based intervention in Tangail district, Bangladesh. Journal of Health Population and Nutrition. 2006; 24:4, 519-529. | Bangladesh | Describe trends in compliance by families with referral when sick newborns were referred by CHWs | Cluster randomized trial | -Families are educated about danger signs -CHWs deliver package of maternal and newborn care interventions at the home, visit home post-partum period to examine newborns and refer if need urgent care | -Increase in proportion of sick newborns for whom care was sought outside the home and from qualified providers -Decrease in the proportion of those who sought care from unqualified providers | CHW linkages to private referral hospital |
| 18 | Basso MB, Nunes NB, Correa LBC, Vieira CN, Vilarinho Jlpd, and Pucca GA. The construction of the oral health care network in the Federal District, Brazil. Ciencia & Saude Coletiva. 2019; 26:6, 2155-2165. doi: 10.1590/1413-81232018246.08552019. | Brazil | -To explain the process of creation of the oral health care network, through a specific institutional actor – the Dental Management of the SHS-DF -Identify elements, actions, strategies that contributed to effectiveness of process | Qualitative case study | Establishment of oral care network at primary level and dental specialty centers at regional hospitals | "change in the paradigm of the work process, characterized by the connection with community and multi-professional performance" | Oral healthcare network |
| 19 | Batson, J. Guiding community-based public health planning in rural New Mexico. Journal of Public Health Management & Practice. 2002; 8:1, 47-52. | USA | Description of program | Program overview | -Community care network groups 25 agencies and 100 individuals on a monthly basis to provide the action framework for community initiated programs -Organization of the Turning Point Initiative through this network | "Through this collaboration, information is shared regarding the focus of agencies, funding for programming is sought through the most appropriate agency, and much of the competitive energy has been channeled into sharing of resources to write the best funding proposal and implement the best program when funding is obtained" | Community care network |
| 20 | Beyeler N, de la Cruz AY, and Montagu D. The impact of clinical social franchising on health services in low- and middle-income countries: a systematic review. PLoS ONE. 2013; 8:4, e60669. | Studies included from Nepal, Myanmar, Kenya, Pakistan, Philippines, Madagascar, Viet Nam, Ethiopia, India | Evaluate the effect of clinical social franchising clients, communities, and private providers, and on healthcare quality, equity, cost-effectiveness, and health outcomes | Systematic review | Social franchises - reproductive health | -Social franchising positively associated with increased client volume and client satisfaction  -Findings regarding health care utilization and health impact were mixed -Poorer outcomes on cost-effectiveness and equity | Social franchise |
| 21 | Bhatta S, Rajbhandari S, Kalaris K, and Carmone AE. The Logarithmic Spiral of Networks of Care for Expectant Families in Rural Nepal: A Descriptive Case Study. Health Systems and Reform. 2020; 6:2. doi:10.1080/23288604.2020.1824520. | Nepal | -Describe to what extent the processes followed to establish, operate, and refine the network of safety model addresses the four domains of NOC;  -Explain the network of safety approach through the network of care framework | Descriptive case study | Network of safety approach: model to improve maternal and neonatal survival, investments in workforce, infrastructure, essential equipment, technical support, training, mentoring, supervision, community engagement, systematic behavior change, program planning and budget management support to local government - which facilitates continuation of activities after OHW support transitions | -Contributed to reductions in preventable maternal and neonatal deaths -End-line surveys in two districts service utilization surpassed the 30% target -Reduction of maternal and newborn deaths by 80%, surpassing the 50% target | Network of Care/network of safety |
| 22 | Bowen JR, Callander I, Richards R, and Lindrea KB. Decreasing infection in neonatal intensive care units through quality improvement. Archives of Disease in Childhood. Fetal and Neonatal Edition. 2017; 102:1. F51–F57. doi:10.1136/archdischild-2015-310165. | Australia | -To decrease the incidence of neonate bloodstream infection through a quality improvement program -Describe QI activities in the program -Describe process of almost real time data to audit infections | Statistical analyses of network data on blood stream infections | Sepsis Prevention in NICUs Group Project - multihospital quality improvement initiative | State-wide QI initiative was effective | Quality improvement network |
| 23 | Bowker SL, Stelfox HT and Bagshaw SM. Critical care strategic clinical network: Information infrastructure ensures a learning health system. CMAJ. 2019; 191(Suppl 1):S22-3. doi: 10.1503/cmaj.190578. | Canada | Overview of Alberta's Critical Care Strategic Clinical Network | Commentary: description of network | Foster a learning health system using provincial informatics infrastructure to drive innovation, implement evidence-informed practice, and evaluate outcomes | Example: sustained improvements in screening and a measurable reduction in delirium-days | Critical Care Strategic Clinical Network made up of 20 provincial ICUs |
| 24 | Britto M, Fuller SC, Kaplan HC, Kotagal U, lannon C, Margolis PA, Muething SE, Schoettker PJ, and Seid M. Using a network organisational architecture to support the development of Learning Healthcare Systems. BMJ Qual Saf. 2018; 27; 937-946. doi:10.1136/bmjqs-2017-007219. | USA | Describe the common framework and methods of Learning Networks and how the network model supports transformation of the health system to a Learning Health System | Framework paper | Design, development, and implementation of network-based Learning Healthcare Systems called Learning Networks | Examples from different networks: -Improvements in care processes and outcomes, which have been sustained, 80% of population in clinical remission -Reduction in interstage mortality by 40% and 95% survival across the network -Reduction in hospital-acquired conditions by 5% to 79% -Improved neonatal outcomes, reduction by 40% of medically unnecessary early delivery | Learning Network: a network-based Learning Health System |
| 25 | Broughton E, Hermida J, Hill K, Sloan N, Chavez M, Gonzalez D, Freire JM, and Ximena Gudino. Evaluation of an intervention to improve essential obstetric and newborn care access and quality in Cotopaxi, Ecuador. Frontiers in Public Health. 2016; 4:247. doi: 10.3389/fpubh.2016.00247. | Ecuador | To evaluate changes in facilities participating in the EONC network with respect to access and quality of EONC and compare to non-participating facilities | Evaluation: household survey at baseline and endline of intervention and control groups, facility chart reviews, simulation care by TBAs to assess knowledge and skills | Network created among health services to improve service provision and provide culturally sensitive, quality EONC services, including transport of obstetric and newborn emergencies | -Percentage of women receiving a postnatal visit within first 2 days of delivery increased  -Postpartum/counselling on newborn care increased  -Increased community and facility quality of care and improved mothers’ health knowledge -Continual decline in newborn mortality in project intervention years | Provincial to community level MNH QI network |
| 26 | Brown BB, Haines M, Middleton S, Paul C, D'Este C, Klineberg E, and Elliott E. Development and validation of a survey to measure features of clinical networks. BMC Health Services Research. 2016; 16:531. doi:10.1186/s12913-016-1800-0. | Australia | "to outline the development, validation and descriptive results of an Internet survey designed to assess the effectiveness of clinical networks in order to guide future strategic and operational management and leadership in the wider context in which they operate" | -Development of survey instrument -Psychometric assessment of survey instrument -Survey of sample of network members | Survey of effectiveness of clinical networks | "network members there was strong reported commitment and belief in network-led quality improvement initiatives, which were perceived to have improved quality of care (72.8 %) and patient outcomes (63.2 %). Network managers were perceived to be effective leaders and clinical co-chairs were perceived as champions for change" | Clinical networks |
| 27 | Brown BB, Patel C, McInnes E, Mays N, Young J, and Haines M. The effectiveness of clinical networks in improving quality of care and patient outcomes: a systematic review of quantitative and qualitative studies. BMC Health Services Research. 2016; 16:360. doi: 10.1186/s12913-016-1615-z. | USA, UK, Canada, Australia | "to ascertain the effectiveness of clinical networks and identify how successful networks improve quality of care and patient outcomes" | Systematic review - quantitative and qualitative studies included | The review focused on managed and non-managed clinical networks | -"clinical networks can be effective vehicles for quality improvement in service delivery and patient outcomes across a range of clinical disciplines" -"variability in the networks’ ability to make meaningful network- or system-wide change in more complex processes" -"networks that had a positive impact on quality of care and patients outcomes were those that had adequate resources, credible leadership and efficient management coupled with effective communication strategies and collaborative trusting relationships" | Clinical networks |
| 28 | Burns E, Collington M, Eden T, Freccero P, Renner L; Paintsil V, Dolendo M, Islam A, Khaing AA and Rosser J. Development of paediatric oncology shared-care networks in low-middle income countries. Journal of Cancer Policy. 2018; 16:26-32. doi: 10.1016/j.jcpo.2018.03.003. | Ghana, Bangladesh | To write up findings from the project as a resource guide, share lessons learned, and make available | Stakeholder workshop: discussions, agreement, defining criteria, exploration, reflection | Establishment of shared-care networks for child cancer are in LMICs | Successes of a shared-care network: Hub (referral center), shared-care center hospitals, good communication, ability to train staff within the network, and supportive hospitals and government | Shared-care network |
| 29 | Carmone AE, Kalaris K, Leydon N, Sirivansanti N, Smith JM, Storey A, and Malata A. Developing a Common Understanding of Networks of Care through a Scoping Study. Health Systems & Reform. 2020; 6:2, e1810921. doi:10.1080/23288604.2020.1810921. | Global | "delineating the concept of NOC and looking at the comprehensive context of interventions, in order to create a common understanding of the term, justify why the approach is necessary, and begin to explore how its use within health systems could be encouraged" | Scoping study: triangulated data from three methods: 1) a scoping literature review; 2) stakeholder perspectives; and 3) descriptive case studies of anecdotally successful operational programs that fit the working definition of NOC | Definition and framework for networks of care | NOC definition and framework | Network of Care |
| 30 | Cassinelli A, Pauselli N, Piola A, Martinelli C, de Azevedo JLA, Bidondo MP, Groisman B, Barbero P, Liascovich R, and Sala A. National Health Care Network for children with oral clefts: organization, functioning, and preliminary outcomes. Archivos Argentinos De Pediatria. 2018; 116;1, E26-E33. doi: 10.5546/aap.2018.eng.e26. | Argentina | Describe network and preliminary outcomes | Descriptive, observational, and cross-sectional study | Development of a care network for newborn infants with oral clefs | 70% of children were assessed by a certified treating institution and were treated | Treatment and referral network for oral clefs |
| 31 | Chan BTB, Rauscher C, Issina AM, Kozhageldiyeva LH, Kuzembaeva DD, Davis CL, Kravchenko H, Hindmarsh M, McGowan J, and Kulkaeva G. A programme to improve quality of care for patients with chronic diseases, Kazakhstan. Bulletin of the World Health Organization. 2020; 98:3, 161-169. doi:10.2471/BLT.18.227447. | Kazakhstan | Describe the results of a disease management program and the effect of the initial design and pilot of the program | Quasi-experimental before/after | "supportive, team based, multifaceted approach to quality improvement that aimed to help clinic teams address the root causes of poor care in an environment that emphasized learning, analysis and improving work processes" | Improvements in care quality process measures | Quality improvement program |
| 32 | Cordier LF, Kalaris K, Rakotonanahary RJL, Rakotonirina L, Haruna J, Mayfield A, Marovavy L, McCarty MG, Aina AT, Ratsimbazafy B, Razafinjato B, Loyd T, Ihantamalala F, Garchitorena A, Bonds MH, and Finnegan KE. Networks of Care in Rural Madagascar for Achieving Universal Health Coverage in Ifanadiana District. Health Systems & Reform. 2020; 6:2, e1841437. doi: 10.1080/23288604.2020.1841437. | Madagascar | Using the NOC framework, describe the innovations and best practices for a NOC in a rural district with limited financial resources | Descriptive case study | Partnership between PIVOT (NGO) and MoH to build a district level model health system based on "integrating clinical care, system readiness, and scientific innovation at all levels of the health system" | -Under-five mortality declined 19% and neonatal mortality by 36% -Care-seeking for childhood illness increased by 51% in PIVOT-supported areas and fell slightly in the rest of the district -Attendance at four or more ANC visits increased for all areas of the district | Network of Care |
| 33 | Cunningham F, Morris A, and Braithwaite J. Experimenting with clinical networks: the Australasian experience. Journal of Health Organization and Management. 2012; 26:6, 685-696. doi: 10.1108/14777261211276961. | Australia | To "draw on the recent extensive development of Australasian clinical networks to identify themes and discuss recurring challenges" | Viewpoint/review | Clinical networks | Various | Clinical networks |
| 34 | Datta V, Srivastava S, and Singh M. Formation of Quality of Care Network in India: Challenges and Way Forward. Indian Paediatrics. 2018; 55:9, 824-827. | India | To provide an overview of the network, its relevance, and impact | Overview of network | "self-sustaining network of facilities implementing Quality Improvement methods for improvement of care" | Self-sustaining network of quality improvement teams, leading the spread and adoption of simple quality improvement strategies across the Indian subcontinent | Nationwide Quality of Care Network |
| 35 | de Almeida PF, de Oliveira SC, and Giovanella L. Network integration and care coordination: the case of Chile's health system. Ciencia & Saude Coletiva. 2018; 23:7, 2213-2227. doi: 10.1590/1413-81232018237.09622018. | Chile | To analyze the implementation of integrated networks and strategies and tools for coordination of PHC care | Exploratory, descriptive-interpretative, and qualitative study | Implementation of integrated networks and tools for coordination of care in Chilean health system through reform and strengthening PHC | Documentation of transition from fragmented to integrated care | Integrated healthcare network |
| 36 | Devarakonda, S. Hub and spoke model: making rural healthcare in India affordable, available and accessible. Rural and Remote Health. 2016; 16:1. | India | To determine if a hub and spoke model can expand market reach and increase profits while reducing costs of operations for organizations and to customers | Exploratory review and interviews | Implementation of hub-spoke model with ICT component | Hub-spoke model with an ICT component is an effective mechanism allowing for greater distribution while minimizing costs | Hub-spoke model |
| 37 | Diallo MM, Diallo AM, Balde NM, Camara A, Balde MC, Kake A, Bah A, Barry TO, Diallo IP, and Lokrou A. Access to diabetes care in sub-Saharan Africa: Results of a diabetes health network in Guinea. Medecine des Maladies Metaboliques. 2013; 7:3, 272-276. | Guinea | Provide an overview of activities of the network and describe its specificities | Program overview | Establishment of diabetes network | -Increase in number of annual consultation (30-fold) -Reduction in hospital death rate (by 80%) | Regional diabetes management network |
| 38 | Diniz PRB, Sales FJR, and Novaes MD. Providing Telehealth Services to a Public Primary Care Network: The Experience of RedeNUTES in Pernambuco, Brazil. Telemedicine and E-Health. 2016; 22:8, 694-698. doi: 10.1089/tmj.2015.0209. | Brazil | Report on the PHC telehealth experience and discuss factors associated with telehealth service utilization | Descriptive study | Implementation of telecare services: teleconsultation, telediagnosis, tele-education, remote screening | -Increase in usage of services, particularly for tele-education, telediagnosis, and tele-consult -Over two-thirds of providers said they changed their referral plans after the consult | PHC-university specialist teleconsultation network |
| 39 | Dixon-Woods M, Bosk CL, Aveling EL, Goeschel CA, and Pronovost PJ. Explaining Michigan: developing an ex post theory of a quality improvement program. The Milbank Quarterly. 2011; 89:2, 167-205. | USA | -"outline an approach, located within the broad family of theory-oriented evaluation methods, for developing ex post theory by using program leaders’ experience and social scientists’ input...describe an application of this approach to the Michigan Keystone Project" -Why was the program successful explain how the program worked on average across participating units | Development of ex post program theory | Quality improvement collaborative: interventions specifically on practices related to CVC care and through promoting cultural and organizational changes regarding safety more generally | -Sustained effects of QI program and showed that there was an associated reduced mortality  median reported infection rate dropped and stayed at the reduced level through the end of the study -The mean rate of infections decreased which was sustained for 18 months after the study | Quality improvement collaborative |
| 40 | D'Mello BS, Bwile P, Carmone AE, Kalaris K, Magembe G, Masweko M, Mtumbuka E, Mushi T, Sellah Z, and Gichanga B. Averting Maternal Death and Disability in an Urban Network of Care in Dar es Salaam, Tanzania: A Descriptive Case Study. Health Systems & Reform. 2020; 6:2. e1834303. doi: 10.1080/23288604.2020.1834303. | Tanzania | To detail the efforts made to build and maintain the CCBRT network of care using the network of care framework | Descriptive case study | -Development of a network of care between 22 government hospitals and catchment facilities -With investments in infrastructure from partners and local government funding -Increased capacity to provide MNH services | -Benefit-cost ratio 6:1 -Redistribution of deliveries from three tertiary facilities (66.1% to 51.6%) to secondary hospitals (34.1%), health centers, and dispensaries (14.4%) -Reduction in hospital delivery volumes with none more that 15,000 deliveries a year (from a high of 16,000 to 22,000/year) -Results of standards-based performance assessments increased from 9% in 2010 to over 75% in 2019 -Reduction in maternal death rate by almost 40%, from 153.8 deaths/100,000 live births in 2012 to 79 deaths/100,000 live births in 2019 -Reduction in fistula cases from over 40/year in 2010 to less than 10/year in 2019 -More variable outcomes in stillbirths and neonatal deaths (stillbirths 29 to 20.5/1000 deliveries from 2013-2019, neonatal deaths 14.48 to 12/1000 live births from 2014-2019) | Network of Care |
| 41 | Dougherty G, Panya M, Madevu-Matson C, Anyalechi GE, Clarke K, Fayorsey R, Kamonga M, Kimambo S, Lutkam D, Mugisha V, Mtiro H, Msuke S, Ramadhani A, Sipemba J, Urasa P, and Rabkin M. Reaching the First 90: Improving Inpatient Pediatric Provider-Initiated HIV Testing and Counseling Using a Quality Improvement Collaborative Strategy in Tanzania. Journal of the Association of Nurses in AIDS Care. 2019; 30:6,682-690. doi:10.1097/JNC.0000000000000066. | Tanzania | The "purpose of our project was to build QI capacity and improve pediatric PITC coverage by designing and supporting a QI Collaborative (QIC) to catalyze swift improvement in PITC performance for pediatric inpatients" | Program overview | Implementation of a quality improvement collaborative for PITC, including interventions on facility-driven change ideas: improvements in staff and client education, staffing patterns, workflow, commodity management, documentation, HIV test kit management, and referrals | -All participating facilities met or surpassed the 80% coverage target for aim 1 at least once and for a median of 5 months -Increase in number of children tested from 38% to 76% -Proportion of children testing positive remained stable -Linkages (aim 2) remained high throughout from 96% to 100% of children linked to care -Overall a 96% increase in number of children living with HIV linked to care -HIV rapid test kits supply improved as stockouts fell from 8.8 days/month to 1.5 days/month -"QICs were used to address a critical need in the pediatric HIV cascade and improve the identification of children living with HIV who had been previously undiagnosed" | Quality improvement collaborative |
| 42 | Duvalko KM, Sherar M, and Sawka C. Creating a system for performance improvement in cancer care: Cancer Care Ontario's clinical governance framework. Cancer Control. 2009; 16:4, 293-302. | Canada | "describes the benefits and results reported by Cancer Care Ontario (CCO) in transforming from a direct provider of cancer services to an organization whose responsibilities include improving the quality of care across the province’s cancer system" | Program overview and case examples | The creation of a clinical governance framework integrating clinical accountability with administrative accountability in an ongoing performance improvement cycle | Examples include: -Increase of specialized surgeries at designated centers -Reduction in 90th percentile surgical waitlist time by 36% and regional variability in waitlist times has decreased -Guideline adherence rates increased from 60% to 77% between 2004-2007 and all regions reporting 100% compliance | Clinical governance and performance improvement system |
| 43 | Elrod JK and Fortenberry JL The hub-and-spoke organization design revisited: a lifeline for rural hospitals. BMC Health Services Research. 2017; 17(Suppl 4):795. | USA | "overview of the hub-and-spoke network and discusses Willis-Knighton Health System’s use of it to establish productive partnerships with rural hospitals" | Program overview | -Development of hub-spoke model and extension to rural hospitals  -Hub provided management leadership and infrastructure support to rural hospitals to act as linked satellite | -The rural hospitals were able to provide more comprehensive services and had greater patient volume than before integration in the network -Benefits to the hub as it opened up a new geography of referred patients that would not have otherwise been in their catchment | Hub-spoke model |
| 44 | English M, Ayieko P, Nyamai R, Were F, Githanga D, and Irimu G. What do we think we are doing? How might a clinical information network be promoting implementation of recommended paediatric care practices in Kenyan hospitals? Health Research Policy and Systems. 2017; 15:4. doi: 10.1186/s12961-017-0172-1. | Kenya | To provide an overview of implementation of CIN strategy, explain how components of the strategy might align with implementation activity typology, explain how the components could cause change using the Behavior Change Wheel, and a proposition of the strategy in realist terms | -Analysis of CIN activities and identify core active components based on typology of implementation components -Use of the Behavior Change Wheel to show to network activities and intervention strategies may lead to change in providers behavior | -Systems oriented intervention focused on the leaders of units -Addressing context and norms influencing practice | Examples:  -Increase in clear documentation of HIV status -Improved uptake of MUAC | Clinical Information Network |
| 45 | Fandre M, McKenna C, Beauvais B, Kim F, and Mangelsdorff AD. Patient-centered medical home implementation effects on emergency room utilization: a case study. Hospital Topics. 2014; 92:3, 59-65. doi: 10.1080/00185868.2014.937967. | USA | "to evaluate the implementation of a PCMH within a high-volume patient utilization area and determine whether those individuals enrolled into a PCMH had a significantly lower likelihood of visiting the ER than those not enrolled" | -Cross-sectional, post-test-only comparison group study -Quasi-experimental  -Explanatory | Implementation of PCMH model for PHC in the military health system | -PCMH enrollees are less likely to visit ER than those registered with a standard PHC clinic -67% reduction in likelihood of utilizing the ER for those registered with a PCMH | Patient centered medical home |
| 46 | Fasawe O, Adekeye O, Carmone AE, Dahunsi O, Kalaris K, Storey A, Osy U, and Wiwa O. Applying a Client-centered Approach to Maternal and Neonatal Networks of Care: Case Studies from Urban and Rural Nigeria. Health Systems & Reform. 2020; 6:2, e1841450. doi: 10.1080/23288604.2020.1841450. | Nigeria | To apply the "NOC framework, the case studies elucidate key elements that made the NOC successful in improving the quality of MNH care and outcomes for women delivering in three states of Northern Nigeria and Lagos Mainland" | Descriptive case study | -Northern Nigeria: MNH program focused on averting preventable deaths in the 24-48 hour window around birth through early identification of potential complications, simple interventions to quickly stabilize and ensure survival, timely and proper referrals to high level care -Lagos State: urban MCH network coordinated from the public sector with public facilities, private sector hospitals, TBA clinics; TBAs linked to formal health sector as a strategy to reduce preventable maternal and neonatal deaths | -Northern Nigeria: reductions in maternal mortality (37%), neonatal mortality (43%), stillbirth (15%), and perinatal mortality (27%) -Lagos: linking TBAs into the formal health system decreased the fragmentation of care for pregnant women and improved referrals; it also increased use of primary care services in Lagos Mainland LGA by an estimated 7% | Network of Care |
| 47 | Fleury MJ and Mercier C. Integrated local networks as a model for organizing mental health services. Administration & Policy in Mental Health. 2002; 30:1, 55-73. | Canada | To describe how integrated service networks came to serve as a model for transforming the mental health system in Québec and to propose a frame of reference | Overview of network | Development of integrated networks for mental health | Outcomes of network not included | Integrated service networks |
| 48 | Foreman S. Montefiore Medical Center in the Bronx, New York: Improving health in an urban community. Academic Medicine. 2004; 79:12, 1154-1161. | USA | To describe Montefiore Medical Centre's experience in building a community-based care system for a large urban population | Case study | Built an integrated system of community-based care | Example: -Implementation of clinical information system led to an improvement in quality and efficiency and reduction in medication errors | Community based case system |
| 49 | Gachau S, Ayieko P, Gathara D, Mwaniki P, Ogero M, Akech S, Maina M, Agweyu A, Oliwa J, Julius T, Malla L, Wafula J, Mbevi G, Irimu G, and English M. Does audit and feedback improve the adoption of recommended practices? Evidence from a longitudinal observational study of an emerging clinical network in Kenya. BMJ Global Health. 2017; 2:e000468. doi:10.1136/bmjgh-2017-000468. | Kenya | "to describe and explore responses to repeated rounds of A&F delivered to 14 facilities in Kenya of performance assessed using indicators representing adoption of or adherence to recommended practices articulated in Kenyan guidelines" | Descriptive and exploratory analyses | -Within the context of the CIN, describe responses to audit and feedback across multiple indicators with different tasks and types of feedback -Examine variations in responses across facilities in the CIN -Examine influence of standard PAR | -Improvements in adoption and adherence to clinical practice recommendations among 70% of analyzed indicators -No indicators had overall deterioration of average performance, though there were site specific declines -Many indicators showed average positive change with time -A PAR linked to audit and feedback showed improved documentation of clinical signs (simple and difficult documentation indicators) | Clinical Information Network |
| 50 | Garcia PJ, Carcamo CP, Garnett GP, Campos PE, and Holmes KK. Improved STD syndrome management by a network of clinicians and pharmacy workers in Peru: the PREVEN Network. PLOS One. 2012; 7:10, e47750. doi: 10.1371/journal.pone.0047750. | Peru | To develop and evaluate an integrated network of physicians, midwives, and pharmacy staff trained in STD syndromic management | Part of a randomized trial | Training of pharmacy workers linked to a referral network of clinicians to manage STD care | -Pharmacies reported more STD cases than clinicians -Improvements in management of STD syndromes at intervention pharmacies | Clinician - pharmacy network |
| 51 | Giessler K, Seefeld A, Montagu D, Phillips B, Mwangi J, Munson M, Green C, Opot J, and Golub G. Perspectives on implementing a quality improvement collaborative to improve person-centered care for maternal and reproductive health in Kenya. International Journal for Quality in Health Care. 2020; 30:10, 671-676. doi:10.1093/intqhc/mzaa130. | Kenya | To understand perspectives and experiences related to participation in a QIC to improve person-centered care for maternal health and FP | Semi-structured qualitative interviews | Quality improvement collaborative focused on improving person-centered care experiences for women using maternal health and FP services | "that sensitization to PCC principles resulted in multiple perceived benefits for staff and patients alike, including improved interactions with patients and clients, deeper awareness of patient and client preferences, and improved interpersonal skills and greater job satisfaction" | Quality improvement collaborative |
| 52 | Gould LJ, Wachter PA, Aboumatar H, Blanding RJ, Brotman DJ, Bullard J, Gilmore MM, Golden SH, Howell E, Ishii L, Lee KH, Paul MG, Rotello LC, Satin AJ, Wick EC, Winner L, Zenilman ME, and Pronovost PJ. Clinical Communities at Johns Hopkins Medicine: An Emerging Approach to Quality Improvement. Joint Commission Journal on Quality & Patient Safety. 2015; 2015; 41:9, 387-95. doi: 10.1016/S1553-7250(15)41050-5. | USA | Overview of clinical communities | Program overview | Establishment of clinical communities across a health network for different health needs | No specific outcomes mentioned | Clinical community for QI |
| 53 | Greene A, Pagliari C, Cunningham S, Donnan P, Evans J, Emslie-Smith A, Morris A, and Guthrie B. Do managed clinical networks improve quality of diabetes care? Evidence from a retrospective mixed methods evaluation. Qual Saf Health Care. 2009; 18, 456-461. doi:10.1136/qshc.2007.023119. | Scotland | To describes the form and impact of quality improvement work in a managed care network for diabetes | Retrospective mixed-methods evaluation | Progressive implementation of quality improvement strategies targeted at individual and clinical teams | -Simple process measures improved quickly -Complex measures improved more slowly and dependent on care pathway redesign | Managed clinical network |
| 54 | Grover TR, Pallotto EK, Brozanski B, Piazza AJ, Chuo J, Moran S, McClead R, Mingrone T, Morelli L, and Smith JR. Interdisciplinary teamwork and the power of a quality improvement collaborative in tertiary neonatal intensive care units. The Journal of Perinatal & Neonatal Nursing. 2015; 29:2, 179-86. doi: 10.1097/JPN.0000000000000102. | USA | "describe the structure and components of the CHND’s new, multi-institutional neonatal QIC, and highlight our inaugural study in which we effectively reduced central line-associated bloodstream infections (CLABSIs) across participating institutions" | Program overview | -Development of a quality improvement collaborative for neonates referred to tertiary NICUs -First QI projected focused on reducing central line-associated bloodstream infections | -CLABSI rates decreased by 20%  -71% of sites in the collaborative participated in the project -Data reporting compliance over 80% | Quality improvement collaborative |
| 55 | Gudlavalleti VSM, Shukla R, Batchu T, Malladi BVS, and Gilbert C. Public health system integration of avoidable blindness screening and management, India. Bulletin of the World Health Organization. 2018; 96:10, 705-715. doi: 10.2471/BLT.18.212167. | India | To provide an overview of two pilot projects on diabetic retinopathy and retinopathy of prematurity and show how elements of the pilots have expanded to other geographies | Program overview | Integrate strategies for control of diabetic retinopathy and retinopathy of prematurity into existing health systems through two pilot programs | Establishment of sustainable and scalable systems for controlling the two conditions that are integrated into the public system and are expanded geographically | Hub-spoke model |
| 56 | Haines MM, Brown B, D'Este CA, Yano EM, Craig JC, Middleton S, Castaldi PA, Pollock CA, Needham K, Watt WH, Elliott EJ, Scott A, Dominello A, Klineberg E, Atkinson JA, Paul C, and Redman S. Improving the quality of healthcare: a cross-sectional study of the features of successful clinical networks. Public Health Research & Practice. 2018; 8:4. doi: 10.17061/phrp28011803. | Australia | To examine factors that make clinical networks effective in improving quality of care and enabling system-wide changes | Retrospective cross-sectional study | -Quantitative assessment of features of clinical networks that influence networks' ability to drive improvements in quality of care and facilitate system-wide change in state-funded clinical networks with a system-wide focus across various disciplines in a large geographic region -Clinicians identify and push for models of service delivery and QI activities | -Three networks had high impact on quality of care -Seven networks had high impact on system-wide change -Better perceived strategic and operational network management led to high ratings on impact of quality of care -Better perceived leadership of network manager and strategic and operational network management associated with higher ratings of impact on system-wide change | Clinical networks |
| 57 | Hansudewechakul R, Naiwatanakul T, Katana A, Faikratok W, Lolekha R, Thainuea V, and McConnell MS. Successful clinical outcomes following decentralization of tertiary paediatric HIV care to a community-based paediatric antiretroviral treatment network, Chiangrai, Thailand, 2002 to 2008. Journal of the International AIDS Society. 2012; 15:17358. doi: 10.7448/IAS.15.2.17358. | Thailand | To assess "factors associated with death and clinical outcomes of HIV-infected children who received care at CRH and CHs after implementation of a community-based pediatric HIV care network" | Quantitative | Tertiary - community hospital provincial level HIC care network of pediatric HIV | Entire cohort -Increase in WAZ score  -Proportion of children moderately and severely underweight decreased from 27% to 1.8% -96-100% reported 95% adherence -CD4 improved from 6% to 26%  -Decrease in proportion of children with severe immune suppression from 80% to 4.8% Subgroup analysis -Children at tertiary or community hospitals in the network had similar adherence levels and numbers of children with VL>400 copies/ml -No difference in rate of weight gain but children from community hospitals had lower WAZ at baseline and throughout the study  -No difference in CD4 gain | Counter-referral network between tertiary hospital and community hospitals |
| 58 | Harrison, L. and Montenegro, G. and Malvares, S. and Astudillo, M. and Behn, V. and Bertolozzi, M. R. and Chiesa, A. M. and Espinoza, M. and Fujimori, E. and Harper, D. and Orellana, A. and Saenz, K. and Sigaud, C. H. and Verissimo Mde, L. The Network for Nursing in Child Health. Pediatric Nursing. 2008; 34:2, 113-6, 138. | South America: Brazil, Chile, Colombia, Argentina | To describe the history and objectives of the Network for Nursing in Child Health | Program overview | Development of a multi-country network for child health nursing, with an initial focus on inclusion of ICMI into nursing curricula | -Augmentation of communication on improving child health among experts -Promotion of use of IMCI guidelines in nursing interventions -Translation of evidence-based protocols for designing interventions in child health -Promotion of student and faculty exchanges -Dissemination of work of the network via publication and presentations -Expansion of knowledge work of nursing, e.g. standardized curriculum and interventions -Development of model for web-based communications | Specialty nursing network |
| 59 | Hilliard LM, Maddox MH, Tang SH, and Howard TH. Development of a regionalized, comprehensive care network for pediatric sickle cell disease to improve access to care in a rural state. Disease Management & Health Outcomes. 2004; 12:6, 393-398. doi: 1173-8790/04/0006-0393. | USA | To evaluate the impact of a rural comprehensive clinical care network | Retrospective analysis | Development of a sickle cell care network | -Percent of newborns identified to have sickle cell enrolled in a clinics increased from 50% to 90%  -Average age at first clinic visit declined from 21 to 5.3 months -Average age of penicillin prescription declined from 4 to 1.1 months -Distance to comprehensive sickle cell care decreased from an average of 90 to 45 miles, increase in percent of patients living within 30 miles of a clinics from 41% to 84%, with 96% living within 60 miles -Death rate due to sepsis decreased from 5.71 to 1.94 deaths/100 patient years | Clinical networks |
| 60 | Hyre A, Caiola N, Amelia D, Gandawidjaja T, Markus S, and Baharuddin M. Expanding Maternal and Neonatal Survival in Indonesia: A program overview. International Journal of Gynecology & Obstetrics. 2019; 144. doi: 10.1002/ijgo.12730. | Indonesia | Provide an overview of the EMAS program | Program overview | To address challenges (high case fatality, unclear referral processes, lack of accountability and reliable data): -Improve clinical governance -Strengthen referral process | Described in other articles in the supplement | Referral networks |
| 61 | Iedema R, Verma R, Wutzke S, Lyons N, and McCaughan B. A network of networks. Journal of Health Organization & Management. 2017; 31:2. doi:10.1108/JHOM-07-2016-0146. | Australia | "to investigate how one agency, the NSW Agency for Clinical Innovation (ACI), and the multiple networks and enabling resources that it encompasses, govern, manage and extend the potential of networks for healthcare practice improvement" | Ethnographic descriptive case study | "government-funded agency that houses a mix of self-steering, government-resourced and government-steered networks which are co-located with “enabler teams” assisting those networks with programme implementation across the State’s health system, with health professional training and programme evaluation" | No specific outcomes mentioned | Network of networks made up of taskforces, institutes, and clinical networks |
| 62 | Irimu G, Ogero M, Mbevi G, Agweyu A, Akech S, Julius T, Nyamai R, Githang’a D, Ayieko P, and English M. Approaching quality improvement at scale: a learning health system approach in Kenya. Arch Dis Child. 2018. doi: 10.1136/archdischild-2017-314348. | Kenya | -To "outline the rationale for and philosophy of the CIN" -"illustrate how the CIN is a mechanism promoting continued improvement of basic hospital services, implementation of new effective practices and technologies, and conduct of locally relevant research to optimise interventions" | Program overview | Development of clinical information network | Examples:  -Increase in proportion of children with PAR from 54.05% to 99.47% -71.5% of children had MUAC recorded measurement | Clinical Information Network |
| 63 | Joseph JP, Jerome G, Lambert W, Almazor P, Cupidon CE, and Hirschhorn LR. Going beyond the vertical: leveraging a national HIV quality improvement programme to address other health priorities in Haiti. AIDS. 2015; 29 (Suppl 2):S165-S173. doi:10.1097/QAD.0000000000000715. | Haiti | To describe the "successful leveraging of expertise and framework of a national HIV quality improvement programme to spread capacity and improve quality across a network of clinics in HIV and other targeted areas of healthcare delivery in rural Haiti" | Program overview and descriptive statistics | Network of facilities supported with infrastructure, HR, training, M&E, EMR for HIV, clinical care, QI | -Facility QI capacity increase with expansion from HIC to other health areas  -Significant increase in capacity of Quality Management Program, performance measurement, quality improvement projects, and patient engagement -Average performance across all HIV indicators increased from 39 to 72% | Quality improvement network |
| 64 | Lannon CM and Peterson LE. Pediatric collaborative networks for quality improvement and research. Academic pediatrics. 2013; 13:6, S69-74. | USA | to "describe the network model, provide examples of these networks in paediatrics, and discuss how paediatric collaborative networks can serve to close the quality gap and accelerate the translation of evidence into practice, resulting in improved care and outcomes for children" | Descriptive review | Pediatric collaborative networks | Examples:  -Quality Transformation Network: Average aggregate CLABSI rate decreased by 56%, saved 355 child lives, over $100m in cost savings -Solutions for Patient Safety: 60% reduction in surgical site infections, 34.5% reduction in adverse drug events, 3576 children avoid adverse events, over $5m in cost savings -CPQCC: reduced central line-associated bloodstream infections, increased breast-feeding rates, achieved normothermia -PPQC: 20% sustained decrease in bloodstream infections and 60% decrease in near-term deliveries without medical indicators  - ImproveCareNow: increase proportion of Crohn's (55% to 68%) and ulcerative colitis (86% to 90%) patients with inactive disease | Collaborative improvement network |
| 65 | Lee SK, Shah PS, Singhal N, Aziz K, Synnes A, McMillan D, and Seshia MM. Association of a quality improvement program with neonatal outcomes in extremely preterm infants: a prospective cohort study. CMAJ. 2014; 186: 13. doi:10.1503 /cmaj.140399. | Canada | "to assess the generalizability of EPIQ to other neonatal units in Canada and to determine any association with improvements in mortality and morbidity among preterm neonates born at less than 29 weeks gestational age" | Prospective cohort study | Evidence-based Practice for Improving Quality (EPIQ): quality improvement approach using the best evidence with institution data to identify institution needs | -Severe retinopathy, necrotizing enterocolitis, and nosocomial infections were significantly reduced -No statistically significant difference in mortality -Most improvement in infants born at 26 to 28 weeks | Quality improvement program |
| 66 | Lee SJC, Higashi RT, Inrig SJ, Sanders JM, Zhu H, Argenbright KE, and Tiro JA. County-level outcomes of a rural breast cancer screening outreach strategy: a decentralized hub-and-spoke model (BSPAN2). Translational Behavioral Medicine. 2017; 7:2. doi: 10.1007/s13142-016-0427-3. | USA | To evaluate the hub-spoke model by quantifying women receiving services at county level and assessing spokes ability to conduct outreach | Mixed methods | Expansion of a hub-and-spoke model for mammography services with local organization outreach | -Average number of women served/month in hub-led and spoke-led counties did not vary  -Similar uptake for screening services among rural, uninsured women despite heterogeneous catchment area -"program expansion was highly successful: the number of women interested in receiving services greatly exceeded program expectations" | Regional hub-spoke model |
| 67 | Lee HC, Bennett MV, Crockett M, Crowe R, Gwiazdowski SG, Keller H, Kurtin P, Kuzniewicz M, Mazzeo AM, Schulman J, Nisbet CC, and Sharek PJ. Comparison of Collaborative Versus Single-Site Quality Improvement to Reduce NICU Length of Stay. Pediatrics. 2018; 142:1, e20171395. doi:10.1542/ peds.2017- 1395. | USA | "to assess the effectiveness of a collaborative QI project designed to optimize LOS for preterm NICU patients" | Evaluation | Collaborative QI model and single-site QI models both implementing evidence-based practice bundle for optimizing length of stay | -Hospitals in the QI collaborative decreased PMA at discharge from 37.8 to 37.5 weeks, decreased in adjusted length of stay from 52.9 days to 50 days, and early PMA at discharge increased from 31.6% of eligible patients to 41.9% -Single NICU QI facilities did not have significant changes in any of the outcome measures  -Non-participant facilities had no significant trend towards overall improvement -Did not see increase in readmission in collaborative QI group | Quality improvement collaborative |
| 68 | Leslie M, Khayatzadeh-Mahani A, Birdsell J, Forest P, G, Henderson R, Gray RP, Schraeder K, Seidel J, Zwicker J, and Green LA. An implementation history of primary health care transformation: Alberta's primary care networks and the people, time and culture of change. BMC Family Practice. 2020; 21:258. doi: 10.1186/s12875-020-01330-7. | Canada | "better understand the PCNs’ implementation history, and so generate a rich, contextualized account of these particular PHC transformation efforts" | Interpretative qualitative study | Implementation of PHC networks | -PCNs shifted from a focus on local solutions to local problems with few rules to a framework of accountability with central demands for standardized measures, governance, and co-planning -A core group of people, time to develop long term QI vision, and understanding and aligning each other's cultures have been central to PCN survival and success | Primary Care Networks |
| 69 | Lin Y. Integration in primary community care networks (PCCNs): examination of governance, clinical, marketing, financial, and information infrastructures in a national demonstration project in Taiwan. BMC Health Services Research. 2007; 7:90. doi:10.1186/1472-6963-7-90. | Taiwan | "to understand the nature and extent of integration to which they and their associated PCCN members (clinics and hospitals) had in governance, clinical, marketing, financial, and information infrastructures" | Descriptive analyses | Demonstration project of Primary Community Care Networks | -"wide variance in the kind and degree of integration among them and a lot of room for better integration" -"PCCNs' members had higher involvement in the governance infrastructure, which was usually viewed as the most important for establishment of core values in PCCNs' organization design and management" -"higher extent of integration of clinical, marketing, and information infrastructures among the hospital-clinic member relationship than those among clinic members within individual PCCNs" -"financial infrastructure was shown the least integrated relative to other functional infrastructures at the early stage of PCCN formation" | Primary Community Care Network |
| 70 | Magill MK, Lloyd RL, Palmer D, and Terry SA. Successful turnaround of a university-owned, community-based, multidisciplinary practice network. Annals of Family Medicine. 2006; 4(Suppl 1):S12-18. doi: 10.1370/afm.54. | USA | To describe the "financial turnaround of a university-owned, community-based, multidisciplinary practice network" | Report | To save a financially failing community-based multidisciplinary practice network | Financial performance improved | Primary Care Networks |
| 71 | Manns BJ, Strilchuk A, Mork M, and Wasylak T. Alberta's Strategic Clinical Networks: A roadmap for the future. Healthcare Management Forum. 2019; 32:6, 313-322. doi: 10.1177/0840470419867344. | Canada | "This article describes the SCNs, their impact to date, and the objectives, areas of focus, and processes Alberta’s SCNs will use to improve health outcomes and health system performance over the next 5 years" | Qualitative | Development and evolution of strategic clinical networks and development of strategic plan building on networks' accomplishments and identifying collaborative areas of focus | No specific outcomes mentioned | Strategic clinical network |
| 72 | Manns BJ and Wasylak T. Clinical networks: enables of health system change. CMAJ. 2019; 25:191, E1299-1305. doi: 10.1503/cmaj.190313. | Canada, England, Scotland, Australia | to "consider evidence of the effect of clinical networks, and discuss potential barriers and enablers of such networks to inform strengthening of health systems in Canada" | Analytical review | Development of clinical networks | Examples:  -Increase in proportion of preterm babies born at hospitals providing highest volume of specialist care (18% to 49%) -Statistically significant improvements in process indicators -Reduction in antipsychotic use from 26.8% to 17.4% -Reduction in length of stay by 1.5 days, complication risk lowered by 11.7%, and increased cost savings -Improved door-to-needle times from 70 to 36 minutes  -143,800 hospital bed days avoided with $62.5m net savings  -Reduction in time interval from referral to initial assessment by the service from 19.5 to 10 days - 37% of networks had moderate impact and 16% high impact 37% of networks had moderate impact and 37% had high impact on facilitating system-wide change | Clinical networks |
| 73 | Martinez B, Ixen EC, Hall-Clifford R, Juarez M, Miller AC, Francis A, Valderrama CE, Stroux L, Clifford GD, and Rohloff P. mHealth intervention to improve the continuum of maternal and perinatal care in rural Guatemala: a pragmatic, randomized controlled feasibility trial. Reproductive Health. 2018; 15:120. doi: 10.1186/s12978-018-0554-z. | Guatemala | To evaluate the impact of an mhealth application on the use of referral by TBAs for higher-level maternal and perinatal medical care for rural patients in Guatemala | RCT | A perinatal monitoring mHealth platform was introduced in the daily practice of participating TBAs in a unidirectional cross-over study design | -Referral rates for pregnancy and childbirth complications were higher among TBAs that had access to mhealth application | TBA referral network |
| 74 | Mate KS, Ngubane G, and Barker PMA quality improvement model for the rapid scale-up of a program to prevent mother-to-child HIV transmission in South Africa. International Journal for Quality in Health Care. 2013; 25:4, 373-380. doi:10.1093/intqhc/mzt039. | South Africa | To describe how NGOs and the National Department of Health jointly developed the Accelerated Plan to improve PMTCT services at facilities in South Africa | Program overview | -Demand-side intervention to increase the number of pregnant women attending ANC and accessing PMTCT interventions -Supply-side intervention to strengthen delivery of PMTCT services at facilities | -Expansion from 5 to 9 subdistricts due to demand -Main achievement of the project: "demonstrate how a systems improvement design could coordinate available resources and speed up implementation of guideline-based PMTCT care. Two notable achievements - rapid buy-in and project leadership by district managers, and collaboration of multiple supporting NGO partners—were responsible for early success of the intervention and provide a potential model for implementation of other large-scale programs in similar resource-constrained settings" | Quality improvement network |
| 75 | McGivern G, Nzinga J, and English M. 'Pastoral practices' for quality improvement in a Kenyan clinical network. Social Science & Medicine. 2017; 195:115-122. doi:/10.1016/j.socscimed.2017.11.031. | Kenya | "to develop a formative explanation and theory of change for the organisational processes underlying network activities, developed through interdisciplinary, insider-outsider dialogue" | Qualitative case study | "promotes and supports development and use of evidence-based guidelines, measurement of clinical activities and outcomes, leadership and a form of QI involving meeting and discussing the practical challenges of delivering QI and locally solving problems highlighted by audit" | "lateral accountability and governance mechanisms, associated with pastoral practices influencing professional status, may provide a means for motivating health care improvement in LMICs" | Clinical Information Network |
| 76 | McInnes E, Middleton S, Gardner G, Haines M, Maertsch M, Paul CL, and Castaldi P. A qualitative study of stakeholder views of the conditions for and outcomes of successful clinical networks. BMC Health Services Research. 2012; 12:49. doi:10.1186/1472-6963-12-49. | Australia | "aims of this study were to identify key stakeholders’ views on the conditions required to establish successful and effective clinical networks and also their views on desirable outcomes of successful networks" | Qualitative | Stakeholders’ perspectives on clinical networks | Did not specify, main findings in characteristics and other section | Clinical networks |
| 77 | McInnes E, Haines M, Dominello A, Kalucy D, Jammali-Blasi A, Middleton S, and Klienberg E. What are the reasons for clinical network success? A qualitative study. BMC Health Services Research. 2015; 15: 479. doi: 10.1186/s12913-015-1096-5. | Australia | "to identify the views of stakeholders internal and external to clinical networks regarding influential factors in NSW clinical networks, in terms of achieving impacts on quality of care and system-wide change during the period 2006–2008" | Qualitative | Stakeholders’ perspectives on clinical networks regarding impacts on quality of care and system-wide change | Did not specify, main findings in characteristics and other section | Clinical networks |
| 78 | McIntosh N, Grabowski A, Jack B, Nkabane-Nkholongo EL, and Vian T. A Public-Private Partnership Improves Clinical Performance In A Hospital Network In Lesotho. Health Affairs. 2015; 34:6, 954-962. | Lesotho | "purpose of this study was to compare measures of capacity, utilization, clinical quality, and patient outcomes before and after the implementation of a health care public-private partnership" | Mixed methods | Private entity partially financed and designed, built, equipped, and operated a new referral hospital and clinic and upgraded three network clinics | -PPP network higher staffing levels -PPP network had increased utilization, while average length of inpatient stay and casualty visits were lower -Improved quality in the PPP network -Decreased rate of mortality and stillbirths and increased rate of c-section in PPP network | Public-Private-Partnership Integrated Healthcare Network |
| 79 | Mullany LC, Lee CI, Paw P, Od EKS, Maung C, Kuiper H, Mansenior N, Beyrer C, and Lee TJ. The MOM Project: Delivering maternal health services among internally displaced populations in eastern Burma. Reproductive Health Matters. 2008;16:31, 44-56. doi: 10.1016/S0968-8080(08)31341-X. | Burma | "describes the rationale for this innovative model of delivering maternal health and family planning services, provides an overview of the programme structure, training and roles of the health care providers in the project, and outlines the planned monitoring and evaluation activities" | Program overview | Pilot a model of integrated package of MNH and FP interventions through a network of tiered mobile health workers | Reported separately | Three-tiered collaborative network of community-based maternal health workers |
| 80 | Murki S, Kiran S, Kumar P, Chawla D, and Thukral A. Quality Improvement Collaborative for Preterm Infants in Healthcare Facilities. Indian Pediatrics. 2018; 55:9, 818-823. | India | To "describe the concept of Collaborative quality improvement, and the success stories of the best-known Collaborative quality improvement initiatives across the world...highlight the process and progress of creating Collaborative quality improvement in our country" | Review and program overview | Neonatal quality improvement collaborative to decrease care associated infections | "the hospital with the highest incidence of healthcare associated infections showed the maximum improvement with more than 50% reduction from baseline in both microbiological and clinical blood stream infections per 1000 patient days" | Quality improvement collaborative |
| 81 | Nahimana E, McBain R, Manzi A, Iyer H, Uwingabiye A, Gupta N, Muzungu G, Drobac P, and Hirschhorn LR. Race to the Top: evaluation of a novel performance-based financing initiative to promote healthcare delivery in rural Rwanda. Global Health Action. 2016; 9:1, 32943, doi: 10.3402/gha.v9.32943. | Rwanda | To evaluate outcomes associated with a PBF capacity-building model throughout one district | Program overview and mixed-effects linear regression | "district-wide PBF scheme to reward health centres upon reaching district-identified targets that reflect district-level health priorities, supported with technical support in quality improvement (QI) and peer-to-peer learning" | -Increase in community-based health insurance from 68% to 93% -Mean number of acute malnutrition cases declined from 24 to 5 per facility -CPR increased from 42% to 59% | PBF linked quality improvement collaborative program |
| 82 | Ortiz-Barrios M and Alfaro-Saiz JJ. An integrated approach for designing in-time and economically sustainable emergency care networks: A case study in the public sector. PLoS ONE. 2020; 15:6, e0234984. doi: 10.1371/journal.pone.0234984. | South America | "to develop an integrated framework based on Discrete-event simulation, lean manufacturing and six sigma techniques for designing in-time ECNs…the creation of a scheme that guarantees the efficient distribution of payments among the ECN participants" | Methodological overview and case study | Development of emergency care network | Average waiting time in an emergency care network can be reduced with the optimized coordination between emergency departments | Emergency Care Network |
| 83 | Payan DD, Sloane DC, Illum J, Vargas RB, Lee D, Galloway-Gilliam L, and Lewis LB. Catalyzing Implementation of Evidence-Based Interventions in Safety Net Settings: A Clinical-Community Partnership in South Los Angeles. Health Promotion Practice. 2017; 18:4, 586-597. doi: 10.1177/1524839917705418. | USA | "demonstrates how a CBO acted as an external facilitator and employed a collaborative partnership model to catalyse implementation of evidence based interventions in safety net settings" | Qualitative process evaluation | Hypertension evidence-based interventions in a clinical safety net setting: "(1) increasing early follow-up of patients, (2) encouraging intensification of therapies, and/or (3) increasing engagement in lifestyle interventions" | Participants said the collaborative improved the quality of patient care and preventive medicine, increased resource awareness, provided a structured plan for sustained improvements | Clinical community |
| 84 | Rabkin M, Achwoka D, Akoth S, Boccanera R, Kimani M, Leting I, Madevu-Matson C, Mutei R, Nyaga L, Onyango C, Ouma C, Rondinelli I, Rumunyu P, Tsiouris F, Wakoli A, Walker L, and Dougherty G. Improving Utilization of HIV Viral Load Test Results Using a Quality Improvement Collaborative in Western Kenya. The Journal of the Association of Nurses in AIDS Care : JANAC. 2020; 31:5, 566-573. doi: 10.1097/JNC.0000000000000158. | Kenya | "to design and implement an QI collaborative (QIC), using well-established QI methods to empower frontline HCW to explore and address the root causes of suboptimal VLT utilization" | Program overview | Quality improvement collaborative focused on improving viral load testing utilization | -All facilities increased the proportion of patients with unsuppressed viral load completing three adherence sessions within 4 months to over 90% (from 40% to 93%) -Switching patients to second line ART with persistent unsuppressed viral load within 2 months from 58% to 84% -Zero reports of stocked out second line | Quality improvement collaborative |
| 85 | Ramos Junior AN, Correia D, Almeida EA, and Shikanai-Yasuda MA. History, current issues and future of the brazilian network for attending and studying Trypanosoma cruzi/HIV coinfection. Journal of Infection in Developing Countries. 2010; 4:11. | Brazil | "to describe the Brazilian response to the challenges of Chagas disease: the history, current issues, and future of the Brazilian Network for attending and studying *T.cruzi*/HIV coinfection" | Descriptive study | Development of a care, research, and coordination network to manage *T/cruzi/*HIV coinfection | -Structure of national technical guidelines and standards, health care and research protocols and resource priorities  -Mobilization and advocacy towards HIV/AIDS reference centers on coinfection occurrences | Care network |
| 86 | Rask K, Naylor D, and Schuessler L. Voluntary Hospitals Coalitions to Promote Patient Safety. Advances in Patient Safety. 2005; 3: 493-505. | USA | "describes a unique patient safety initiative developed by the Georgia Hospital Association (GHA), working closely with State regulatory agencies and health care professional groups" | Program overview | Patient safety program | -"engaging health care organizations in an iterative change process, while focusing on an ultimate goal, can yield promising results" -"Most hospitals report significant improvement following implementation of their improvement plans, but not all do" | Partnership for Health and Accountability |
| 87 | Rossiter M, Verma J, Denis JL, Samis S, Wedge R, and Power C. Governing Collaborative Healthcare Improvement: Lessons From an Atlantic Canadian Case. International Journal of Health Policy & Management. 2017; 6:12, 691-694. doi:10.15171/ijhpm.2017.60. | Canada | Examines an approach to governing collaborative health system improvement focused on governance structure, governance capacity, and sustaining improvement | Program overview | Quality improvement collaborative focused on chronic diseases | -Spread of PEER model -Symposium to share and disseminate success in intervention implementation -Development of diabetes registry, enhance skills, and education of clinicians in diabetes self-management support | Quality improvement collaborative |
| 88 | Salamanca O, Geary A, Suarez N, Benavent S, and Gonzalez M. Implementation of a diabetic retinopathy referral network, Peru. Bulletin of the World Health Organization. 2018; 96: 674-681. doi: 10.2471/BLT.18.212613. | Peru | "To describe the implementation of a diabetic retinopathy referral network incorporating all levels of health care" | Program overview | Establishment of referral network of primary, secondary, and tertiary facilities for diabetic retinopathy screening and treatment | -138.1% increase in diabetic retinopathy screenings -Screening at secondary facilities increased and reduced at tertiary level | Referral networks |
| 89 | Schneider H, George A, Mukinda F, and Tabana H. District Governance and Improved Maternal, Neonatal and Child Health in South Africa: Pathways of Change. 2020; 6:1, e1669943, doi: 10.1080/23288604.2019.1669943. | South Africa | "this paper seeks to shed light on the value and potential of a governance lens on district health system strengthening for improved health outcomes" | Independent retrospective evaluation, qualitative case study | Introduction of a new district and sub-district governance mechanism - Monitoring and Response Unit | -Improvement in cause-specific under 5 deaths, particularly for severe acute malnutrition -Reduction in number of maternal deaths - by half in one district -However difficult to attribute these changes directly to the intervention -Changes in scope, quality, and organization of MNCH services attributed to introduction of the MRU with the support of DCST and program managers -Enhanced community and PHC screening, improve referral systems across levels of care and between clinicians, improvement in clinical practices in hospitals, better continuity of care | Monitoring and Response Unit |
| 90 | Sheaff R, Benson L, Farbus L, Schofield J, Mannion R, and Reeves D. Network resilience in the face of health system reform. Social Science & Medicine. 2010; 70:779-86. doi: 10.1016/j.socscimed.2009.11.011. | UK | "To examine how quasimarket reforms impact upon health networks’ macroculture" | Narrative case studies | Four networks: child mental health network, self-care network, sub-regional cardiac heart disease network, small local cardiac heart disease network | -"reforms stimulated production of managerial artefacts, changed the function of two networks into commissioning support and in three networks brought the networks’ espoused values closer to national policy, especially for evidence-based practice" -"quasi-market reforms did therefore alter the existing macroculture in three of the networks, but did not radically conflict with it" -"network macrocultures adapted through a sequence of changes" -"core artefact production as the activity driving the development of the other aspects of network macroculture" | Clinical networks and professional networks |
| 91 | Shortell SM and Gillies R. United States innovations in health care delivery. Public Health Reviews. 2010; 32:1, 190-212. | USA | Discussion of three new organizational forms of care - PCMH, ACO, and PHMS - that may be able to respond to new payment incentives | Review | Implementation of PCMH, ACO, and PMHS | -PCMH: improvements in quality of care, patient satisfaction, care coordination, and access to care leading to reduction in ER visits and hospitalizations at same or lower cost (various specific examples cited) -ACO: more integrated forms provide better care at same or lower cost per capita (various specific examples cited) | PCMH linked to ACO linked to PMHS |
| 92 | Sibbald S, Schouten K, Sedig K, Maskell R, and Licskai C. Key characteristics and critical junctures for successful Interprofessional networks in healthcare - a case study. BMC Health Services Research. 2020; 20:700. doi: 10.1186/s12913-020-05565-z. | Canada | -"describes lessons learned from the successful implementation of a small localized primary care network to support the development and successful implementation of similar networks in other geographical locations" -Explore network complexity | Exploratory case study | Development of Primary Care Innovation Collective and implementation of their Lung Health Program | -Improving quality of life of over 2500 patients by 90% -Provided better patient outcomes at a lower cost | small local network: Primary Care Innovation Collective |
| 93 | Sloan NL, Storey A, Fasawe O, Yakubu J, McCrystal K, Wiwa O, Lothe LJ, and Grepstad M. Advancing Survival in Nigeria: A Pre-post Evaluation of an Integrated Maternal and Neonatal Health Program. Maternal and Child Health Journal. 2018; 22:986-997. doi: 10.1007/s10995-018-2476-3. | Nigeria | "to evaluate the MNH program impact on reducing women’s, neonatal and perinatal mortality and stillbirth" | Pre-post design | Holistic integrated approach to improving MNH by addressing critical gaps in care through linking the health system from household to hospital | -MMR declined 37%, NMR declined 43%, stillbirth rates declined 15%, PMR declined 27% | Integrated maternal and neonatal health program/Network of Care |
| 94 | Spence K and Henderson-Smart D. Closing the evidence-practice gap for newborn pain using clinical networks. Journal of Paediatrics and Child Health. 2011; 47:92-98. doi:10.1111/j.1440-1754.2010.01895.x. | Australia | "aim of the project was to identify gaps between evidence and practice and to introduce strategies within a model to close the gaps" | Evaluation | Implementation model of research utilization: "examining the available evidence, baseline surveys of current practices, providing educational workshops on critical appraisal, examining the specific contexts in relation to organisational support and team involvement in decision making" | -21% overall improvement in number of infants receiving breastfeeding or sucrose for procedural pain -Use of pain assessment tool increased from 14% to 22% -56% of units introduced use of the tool -Improvement in proportion of families aware of infant pain and strategies to manage from 19% to 57% | Clinical networks |
| 95 | Srivastava S, Datta V, Garde R, Singh M, Sooden A, Pemde H, Jain M, Shivkumar P, Bang A, Kumari P, Makhija S, Ravi T, Mehta S, Garg BS, and Mehta R. Development of a hub and spoke model for quality improvement in rural and urban healthcare settings in India: a pilot study. BMJ Open Quality. 2020; 9:e000908. doi: 10.1136/bmjoq-2019-000908. | India | To evaluate the feasibility of employing a hub-and-spoke model for QI in rural and urban settings in India with support from a QI network | Evaluation | Implementation of QI initiatives through a hub-and-spoke model with mentor component | -10 QI projects completed by teams -34 mentoring visits completed by network mentors (14 planned) -Rural hub-mentors completed 4 visits (18 planned) | Quality improvement network |
| 96 | Syengo M and Suchman L. Private Providers' Experiences Implementing a Package of Interventions to Improve Quality of Care in Kenya: Findings From a Qualitative Evaluation. Global Health-Science and Practice. 2020; 8:3, 478-487. doi: 10.9745/GHSP-D-20-00034. | Kenya | -"examines private providers’ experiences with a package of interventions intended to improve the quality of small and medium-sized private health facilities in Kenya" -"seek to better understand private providers’ experiences in this context to determine whether providers felt their clinical quality improved through participation in a comprehensive package of quality improvement interventions, the challenges they faced, and what other opportunities might exist for improving health care quality in Kenya" | Qualitative Evaluation | Increase access to high-quality primary care for low-income clients through a comprehensive package of QI interventions | -"engaging private providers in efforts to improve quality of care in private clinics through a package of interventions that extend beyond the typical social franchising model is achievable" -"costs of implementing a comprehensive quality improvement program remained a critical concern for private providers" | Social franchise |
| 97 | Tayler-Smith K, Zachariah R, Manzi M, Van den Boogaard W, Nyandwi G, Reid T, De Plecker E, Lambert V, Nicolai M, Goetghebuer S, Christiaens B, Ndelema B, Kabangu A, Manirampa J, and Harries AD. An ambulance referral network improves access to emergency obstetric and neonatal care in a district of rural Burundi with high maternal mortality. Tropical Medicine & International Health. 2013; 18:8,993-1001. doi:10.1111/tmi.12121. | Burundi | " (i) describe the communication and ambulance service together with the cost; (ii) examine the association between referral times and maternal and early neonatal deaths; and (iii) assess the impact of the referral service on coverage of complicated obstetric cases and caesarean sections" | Cross-sectional study, retrospective analysis | EmONC referral facility linked to ambulance and communication referral system | -Median time from call to ambulance dispatch 30 min -Median referral time from call to ambulance arriving at referral facility with patient 78 min -Yearly costs 61 Euro/obstetric case; 0.43 Euro/capita/year based on area population -1 maternal death and 104 early neonatal deaths (10%) -Referral times of > 3h associated with higher risk of neonatal death (15% vs. 9%) | Referral networks |
| 98 | Vazquez ML, Vargas I, Unger JP, Mogollon A, Silva MR, and Paepe PD. Integrated health care networks in Latin America: toward a conceptual framework for analysis. Pan American Journal of Public Health. 2009; 26:4, 360-7. | Latin America | "This paper describes different types of IHN that are found in the international context, discusses their risks and benefits, and develops a conceptual framework for their analysis" | Literature review | Introduction of integrated healthcare networks in health system reform in Latin America | -Development of a conceptual framework: analyzing network performance, taking into account internal processes developed by the networks to achieve their objectives, and context of the network -Intermediate outcomes: coordination, continuity of care, access -Final outcomes: equity of access and efficiency | Integrated healthcare network |
| 99 | Vergara MTM, de Vera EA and Carmone AE. Building Trust to Save Lives in a Metro Manila Public-Private Network of Care: A Descriptive Case Study of Quirino Recognized Partners in Quezon City, Philippines. Health Systems & Reform. 2020; 6:2, e1815473, doi: 10.1080/23288604.2020.1815473. | Philippines | To describe the Quirino Respect Partners program using the Network of Care framework | Descriptive case study | Quirino Recognized Partners: linking tertiary public hospital with private and public midwifery clinics to better manage low and high-risk deliveries by decongesting the maternity ward at the tertiary hospital | -Increase in early inward referrals for higher risk cases -Impact on reduction of maternal mortality: in 2014 QRP MMR 49% lower than the city (35/100,000) -Improvement in neonatal mortality but not statistically significant (44.25/1000 to 32/1000) -Observed decline in incidence of perinatal asphyxia -"Midwives in the lying-in clinics in the QRP network have expressed deep satisfaction with being in QRP and increased confidence in their practice; they also report higher (sometimes doubled) income as a recognized partner" | Network of Care |
| 100 | Waiswa P, Manzi F, Mbaruku G, Rowe AK, Marx M, Tomson G, Marchant T, Willey BA, Schellenberg J, Peterson S and Hanson C. Effects of the EQUIP quasi-experimental study testing a collaborative quality improvement approach for maternal and newborn health care in Tanzania and Uganda. Implementation Science. 2017; 12:89. doi: 10.1186/s13012-017-0604-x. | Tanzania, Uganda | "report the effect of the EQUIP intervention on the coverage and quality of essential maternal and newborn health care interventions and knowledge of danger signs after 15 and 26 months of full implementation in Uganda and Tanzania" | Quasi-experimental, plausibility design | QI approach using collaborative model of improvement in two districts at district, facility, and community level, compared to two non-intervention districts using continuous household and facility surveys | -In Tanzania increase in proportion of women with a live birth receiving uterotonics by 26 percentage points -In Uganda increase was 8% -No evidence of association between intervention and immediate breastfeeding or knowledge of maternal and newborn danger signs -In Tanzania some evidence of association between intervention and birth preparedness via clean birth kits for home delivery -In Tanzania some association between the intervention and improvement in supervision visits by district managers to PHC | Quality improvement collaborative |
| 101 | Wang X, Sun XZ, Gong FF, Huang YX, Chen LJ, Zhang Y, and Birch S. The Luohu Model: A Template for Integrated Urban Healthcare Systems in China. International Journal of Integrated Care. 2018; 18:4, 3, 1-10. doi: 10.5334/ijic.395. | China | -This paper studies "the integration process, analyse the core mechanisms, and conduct preliminary evaluations of integrated policy development in the Luohu model" -"aims of this study are to introduce the Luohu model, to evaluate its effects and to explore lessons learned" | Policy paper | "tiered health care delivery system in accordance with a People-Centred Integrated Care model" | -Increase in asset value -Number of GPs doubled, increase in public health physicians -Increase in proportion of hospitalizations going to group hospitals, reducing the cost of health insurance in the group -Increase in proportion of outpatient visits in community health stations from 29.49% to 42.60% -Increase in patients with chronic disease and mental health issues enrolled in case management  -Decrease in pneumonia cases  -High resident satisfaction of community health stations  -Cost of healthcare per resident increase from $675.3 to $844.2  -42.6% of residents saw community health stations as first contact for health services  -Demands of residents drive reforms | Integrated healthcare network |
| 102 | White DE, Virk N, Jackson M, Stelfox HT, Wasylak T, and Ghali W. Experimenting with Governance: Alberta's Strategic Clinical Networks. Healthcare Quarterly. 2019; 21:4, 37-42. | Canada | "examine the rational and potential of this governance intervention, while also considering some of the fundamental questions around their potential impact and the ultimate need for multidimensional assessment" | Review | Implementation of strategic clinical networks | Various, for example: -Additional 1,100 people received stroke care not previously available, saving 3,377 acute care bed days, 88% of patients received early stroke rehabilitation assessments within 48h, 29% decrease in long-term care admissions, satisfaction levels of 92% patients and 97% providers | Strategic clinical network |
| 103 | Willis CD, Riley BL, Herbert CP, and Best A. Networks to strengthen health systems for chronic disease prevention. American Journal of Public Health. 2013; 103:11, e39-48. | Canada | "We use examples from the Canadian Heart Health Initiative and Alberta’s Primary Care Networks to illustrate characteristics of networks, describe the limitations of existing frameworks for assessing the performance of prevention-oriented networks, and propose a research agenda for guiding future efforts to improve the performance of these initiatives" | Review | Service delivery and public health networks | Various outcomes at community, organizations, and network level | Service delivery and comprehensive multilevel public health network |
| 104 | Wood SJ, Albertson EM, and Conrad DA. Accountable Care Program Implementation and Effects on Participating Health Care Systems in Washington State: A Conceptual Model. Journal of Ambulatory Care Management. 2019; 42:4, 321-336. doi: 10.1097/JAC.0000000000000302. | USA | "The purpose of this study was to assess the suitability of an evidence based conceptual model to explain the perceived effects of ACP implementation on participating health care systems in Washington State" | Qualitative comparative case studies | Five different ACO networks and relevant theory | No specific outcomes mentioned | Accountable Care Networks |
| 105 | Yiu V, Belanger F, and Todd K. Alberta’s Strategic Clinical Networks: Enabling health system innovation and improvement. CMAJ. 2019; 4:191(Suppl 1):S1-3. doi: 10.1503/cmaj.191232. | Canada | Brief overview of SCNs and introduce articles in the supplement | Introduction to special edition | Establishment of strategic clinical networks | -Provided a return on investment  -Improvements resulted in 143,800 hospital bed days avoided -Contributed to patient care, safety, and experience, improved health outcomes, developed clinical pathways, collaborator in clinical research | Strategic clinical network |
| 106 | Patel S, Awoonor-Williams JK, Asuru R, Boyer C, Yepakeh Tiah JA, Sheff MC, Schmitt ML, Alirigia R, Jackson EF and Phillips JF. Benefits and Limitations of a Community-Engaged Emergency Referral System in a Remote, Impoverished Setting of Northern Ghana. Global Health: Science and Practice. 2016; 4:4, 552-567. doi: 10.9745/GHSP-D-16-00253. | Ghana | "article provides a summary of the initiative components and evaluates the effectiveness of the program using results from mixed-methods implementation research" | Mixed methods | Establishment of a community and subdistrict level low-cost emergency referral transport-communication system with community education interventions (named SERC (Sustainable Emergency Referral Care)) | -"community exposure to SERC was associated with an increased volume of emergency referrals, diminished reliance on primary care facilities not staffed or equipped to provide surgical care, and increased caseloads at facilities capable of providing appropriate acute care" -"referring facilities often failed to alert receiving facilities of incoming patients, not all patients transported were accompanied by a health worker, and receiving facilities commonly failed to provide patient outcome feedback to the referring facility" | Emergency Referral Care Network |
|  | **Grey literature** |  |  |  |  |  |  |
| 107 | USAID/Engender Health. A collaborative network to improve access to fistula treatment in Nigeria. (Fistula Care). 2010. | Nigeria | Program activities overview | Brief | Development of a clinical peer-support network for fistula with periodic pooled repair and quarterly professional retreats to discuss clinical issues | 958 repair surgeries through pooled events | Clinical peer-support network |
| 108 | Save the Children. The Regional Learning Network: A Model for Improving Maternal and Newborn Health Care Outcomes in Uganda. | Uganda | Program activities overview | Report | Development of a regional learning network based on QI and referral systems | -RLN improved facility readiness to support obstetric and newborn services -Increase in availability of basic newborn care units -Reduced drug and supply stockouts -Increased provide knowledge and confidence in key competencies -Improved documentation of care, including 74% increase in recording of management of premature deliveries -Increase in quality and coverage of newborn care practices, including early initiation of breastfeeding from 12% to 81%, KMC initiative from 7% to 65%, weighing at birth from 25% to 100%, provision of Vitamin K increased by 80% -Skills labs and learning session found as more beneficial  -Improved facility communication -Average early institutional NMR decreased from 1% to 0.5% and stillbirths from 3% to 1.4% -Increase in provider knowledge of indications for newborn referral from 43% to 80% | Regional Learning Network |
| 109 | USAID/Save the Children. USAID's MaMoni Maternal and Newborn Care Strengthening Project: Project Brief. 2019. | Bangladesh | "to advance learning, expand and scale up effective Maternal and Newborn Care (MNC) interventions to substantially improve outcomes for mothers and newborns in Bangladesh" | Brief | -Improve responsiveness of district to deliver patient-centered MNC  -Improve quality of service and governance of quality of care -Sustain improvements in access and demand for services and household practices -Improve national capacity to deliver quality MNC services at scale | No specific outcomes mentioned | District Learning Network for Quality of Care |
| 110 | USAID. Establishing Model Referral Networks in Haiti: MCSP Case Study. 2018. | Haiti | Program activities overview | Brief | Creation of model referral networks | -4406 referrals -1104 counter-referrals -70% of providers followed guidelines on referral registration -87% of patients satisfied with care -53% of providers followed all communication protocols -51% followed all transport protocols -Only 8% of providers often and only 3% always received counter-referral slips -65% of referrals to hospitals within network -Ambulance only transported 26% of referrals | Referral network |
| 111 | Corbett E, Guenther T, and Sitrin D. Facilitated Referral for Newborns with Danger Signs: The role of community health workers. 2013. | Malawi, Nepal, Bangladesh, Uganda | To examine the role of CHWs in identification and referral of newborns with danger signs | Report | Implementation of CHW referral network for newborn care | -Women received home visit during pregnancy: 90% Bangladesh, 97% Nepal, 68% Uganda, 37% Malawi -Women and newborn received home visit within first week of life: 64% Bangladesh, 62% Nepal, 63% Uganda, 17% Malawi -Good knowledge of newborn danger signs and care for newborn complications in Malawi and Nepal | CHW referral network |
| 112 | Federal Ministry of Health Ethiopia. Catchment Based RMNCAH Mentoring: Participant Manual. Version 1.02. | Ethiopia | Implementation of catchment based RMNCAH mentoring | Manual | Catchment-based mentoring | Intervention manual, no outcomes included | Catchment-based mentoring |
| 113 | Ferlie E, Fitzgerald L, McGivern G, Dopson S, Exworthy M, Addicott R, Bennett C, Ceppi M, and Griffin R. Networks in healthcare: a comparative study of their management, impact and performance. Report for the National Institute for Health Research Service Delivery and Organisation Programme. 2010. | UK | To assess the "nature and impact of network forms in the English National Health Service (NHS) and provide evidence to inform future policy choices" | Report | Public policy networks | -"networks often worked on cross cutting objectives across agencies only realistically achievable over the long term" -"increasing co production and influence from users and citizens" -tackled challenging behavior change objectives  -"continuing obstacles to the transfer of information electronically across agency boundaries and only incremental moves to shared ICTs or data bases" -ICTs not a major driver of organizational transformation towards a network and lack of clear network wide knowledge management strategies  -Limited inter-organizational learning across the network examples; poorly developed cross boundary processes; power inequalities, organizational cliques -"widespread shift from a line managerial bureaucratic style of management to a more value driven form of lateral leadership. The absence of middle level general management is striking. It has been supplanted by various local leadership configurations, supplemented by framework setting from the centre"  -Important role of clinical managerial hybrids who link management and clinical worlds | Various academic/research, managed, clinical |
| 114 | Ferlie E, Fitzgerald L, McGivern, Dopson S, and Bennett C. Public Policy Networks and ‘Wicked Problems’: A Nascent Solution. 2011. |  | to "explore the nature and functioning of eight different public policy networks" | Paper |  |  |  |
| 115 | IHI. Breakthrough Series White Paper. 2003. | USA | "to share with readers the problems IHI is working to address; the ideas, changes, and methods we are developing and testing to help organizations make breakthrough improvements; and early results where they exist" | Paper | Development of breakthrough series collaborative | Example for the first collaborative:  -15% of facilities reduced c-section rate by at least 25% -50% of facilities had reductions of 10% to 25% | Breakthrough Series Collaborative |
| 116 | Kirunda R, Rakhmanova N, Wynne L, Mubiru F, Nabaliisa J, Kim C, and Nampewo E. Strengthening Community-based Family Planning Systems through Collaborative Improvement in Busia District, Uganda. Issue 2. 2017. | Uganda | Program activities overview | Brief | Learning site for Community-based FP (CBFP) | -VHTs reported following all steps on in FP counselling checklist with 90% of clients in pilot sites and 60% to 80% in scale-up sites -VHTs increased time counselling clients -Less than 14% of women counselled with partners -Increase in the number of men reached with FP information and counselling from 50 to 300/month -VHTs counselled an average of 149 men/month -Client return rate in all sites 60% to 70% | Service delivery network |
| 117 | Kirunda R, Mubiru F, Akumu E, Wynne L, Rakhmanova N, and Kim C. Applying a Quality Improvement Model to Strengthen Community-based Family Planning Services in Busia District, Uganda. Issue 1. 2016. |  |  | Brief |  |  |  |
| 118 | Manga L and Bagayoko M. Piloting a new approach for capacity building in entomology and vector control at the level of national malaria control programmes. African Health Monitor, Issue 18: Health Systems and Disaster Preparedness and Response, WHO AFRO. 2013. | Cameroon, Kenya, Madagascar, Mali, Mozambique, Senegal, Tanzania | "outline the impressive results of the project and its wider implications for adopting similar approaches across the region" | Report | Development of a professional expert network on vector control | -Set up or strengthened basic capacities in malaria entomology and vector control -Trained 300 national entomologists (basic and advanced) -Increase in the number of field entomology staff | African Network on Vector Resistance to Insecticides |
| 119 | Montagu D, Prata N, Campbell MM, Walsh J, and Orero S Kenya: reaching the poor through the private sector - a network model for expanding access to reproductive health services. Health, Nutrition and Population Discussion Paper, World Bank. 2005. | Kenya | To determine if it is possible to offer RH services through a network of private sector for profit providers without exacerbating inequity in service access | Paper, survey | Provision of reproductive health services through a private sector network | -The network does not exacerbate inequities in access to health services in rural communities  -Clients more likely to be rural and less educated | Private provider network (Kisumu Medical and Educational Trust (KMET)) |
| 120 | Roy T, Marcil L, Chowdhury RH, Afsana K, and Perry H. The BRAC Manoshi Approach to initiating a maternal, neonatal and child health project in urban slums with social mapping, census taking, and community engagement. | Bangladesh | A guide for program leaders to implement effective maternal, neonatal, and child health program in urban slums and to advocate to policy makers the efficacy of these techniques to be widely adopted | Manual | Community-based MNCH program with social mapping, census taking, and community engagement as key strategies | Increase in percentage of deliveries occurring in facilities and declines in MNM in the service areas | CHW referral network |
| 121 | Brun M, Monet JP, Moreira I, Agbigbi Y, Lysias J, Schaaf M, and Ray N. Implementation manual for developing a national network of maternity units - Improving Emergency Obstetric and Newborn Care (EmONC), United Nations Population Fund (UNFPA), 2020. | Global | "This manual aims to share an innovative approach implemented in several countries with a high burden of maternal and newborn mortality and morbidity, each with widely differing contexts, to develop their national network of referral maternity facilities" | Manual | Development, implementation, and monitoring of a national EmONC network | Examples from pilot countries:  -Burundi: decrease in number of designated EmONC health facilities (152 to 112) only slight decrease in population coverage from 72% to 68% within 2 hours travel time -Senegal: decrease in number of designated EmONC health facilities (173 to 142) increase in population coverage from 91% to 92% within 2 hours travel time, increase in number of functioning EmONC facilities from 15 to 29, increase in availability of Magnesium Sulphate and IUDs, increase in number performing vacuum extraction -Togo: decrease in number of designated EmONC health facilities (109 to 67) with population coverage static at 81% within 1 hour travel time | National network for EmONC facilities |
| 122 | WHO QoC Network Brief. 2018. | Global | -Documents detail how the network was conceived and its implementation approach to quality of care for MNCH -Outlines strategic objectives and M&E framework -Reports on progress towards the network strategic objectives and insights into successes and challenges in an effort to inform implementation at scale within the network and beyond | Brief | Implementation of a quality of care network | -"Network has been successful in keeping quality of care at the top of countries’ national health agendas" -Network countries have made efforts to adopt and adapt the WHO MNH quality of care standards to their context and priorities -Active engagement from MoH and partners towards preparing districts and facilities for quality of care implementation, measurement, monitoring, and learning | Network for improving quality of care for maternal, newborn, and child health |
| 123 | WHO QoC Network Brief. 2019. |  |  | Brief |  |  |  |
| 124 | WHO QoC Network M&E Framework. 2019. |  |  | Manual |  |  |  |
| 125 | Quality, equity, dignity: the network to improve quality of care for maternal, newborn and child health – strategic objectives. Geneva: World Health Organization; 2018. |  |  | Report |  |  |  |
| 126 | The network for improving quality of care for maternal, newborn and child health: evolution, implementation and progress. 2017-2020 report. Geneva: World Health Organization; 2021. |  |  | Report |  |  |  |
| 127 | Yang R, Poon MC, Luke KH, Zhao Y, Sun J, Wang X, Wu R, Chen L, Zhang X, and Wu J. Building a network for hemophilia care in China: 15 years of achievement for the Hemophilia Treatment Center Collaborative Network of China. Blood Advances. 2019; 3, Suppl 1, Global Capacity-Building Showcase. | China | Program activities overview | Report | Creation of a treatment center collaborative network for Hemophilia | "great achievements in developing infrastructure, improving health coverage, creating in formation registries, providing professional education, and ensuring cooperation among various organizations" | Hemophilia Treatment Centre Collaborative Network of China |
| **Update searches** | | | | | | | |
| 128 | Pittalis C, Brugha R, Bijlmakers L, Cunningham F, Mwapasa G, Clarke M, Broekhuizen H, Ifeanyichi M, Borgstein E, Gajewski J. Using Network and Complexity Theories to Understand the Functionality of Referral Systems for Surgical Patients in Resource-Limited Settings, the Case of Malawi. International Journal of Health Policy and Management. 2021; 1-12. doi:  10.34172/ijhpm.2021.175. | Malawi | -To explore the referral system for surgical patients  -Unpack how the different aspects of the system and behavior of providers contribute to its functioning | Mixed Methods – surveys, interviews, referral database | Surgical referral network | -Obstacles to referral system functioning – weaknesses in formal coordination structures  -Deficiencies in informal relationships  -Poor systems functioning impacted quality, efficiency, and safety of patient referral-related care | Surgical referral network |
| 129 | Lopez-Vazquez J, Perez-Martinez DE, Vargas I, Vazquez ML. Interventions to Improve Clinical Coordination between Levels: Participatory Experience in a Public Healthcare Network in Xalapa, Mexico. International Journal of Integrated Care. 2021;21 (4): 12,1-17. doi: 10.5334/ijic.5892. | Mexico | “To analyse the factors that influence the implementation of participatively designed interventions and their effects on clinical coordination between levels of care in a public health network” | Qualitative, descriptive-interpretative study | -Two participatory action research cycles to design and implement the intervention  -Offline virtual consultations between primary and secondary care physicians  -Joint training meetings between levels of care | Clinical coordination between levels of care was improved by involvement in designing the interventions, institutional support, and reflexive methods for training | Public healthcare network |
