# Supplementary material for: Understanding networks in low-and middle-income countries’ health systems: A scoping review
Source: PLOS Glob Public Health. 2023 Jan 11;3(1):e0001387. doi: 10.1371/journal.pgph.0001387 (PMC10022031; doi:10.1371/journal.pgph.0001387)
Supplement: S3 Appendix — (DOCX) [file pgph.0001387.s007.docx]

## S7 Appendix. Reported Network Components and Characteristics

**Table 1:** The component form and structure with the corresponding component characteristics and the reference numbers of the selected literature for each characteristic. Number of different published, grey, and total literature sources is included for each characteristic.

| **Form and Structure** -Creation of the network: top-down/mandated vs. bottom-up/organic -Linkages between entities -Existence of a network coordinating entity | **Publication Number (published + grey literature)** | **# Published** | **# Grey** | **Total #** |
| --- | --- | --- | --- | --- |
| connecting across levels of the health system and entities | 27, 59, 1, 36, 43, 66, 4, 3, 21, 32, 46, 93, 99, 91, 2, 7, 38, 89, 22, 95, 11, 13, 30, 57, 60, 97, 28, 6, 17, 35, 47, 48, 68, 69, 79, 101, 10, 82, 109, 112, 127, 128 | 39 | 3 | 42 |
| established vision, mission, shared values, targets, rules, roles, responsibilities, culture | 62, 27, 33, 71, 72, 90, 102, 105, 42, 61, 15, 24, 53, 21, 32, 40, 99, 19, 91, 96, 89, 104, 12, 41, 52, 80, 84, 87, 60, 48, 70, 92, 101, 82, 85 | 35 |  | 35 |
| network agreements / network mapping | 61, 55, 32, 40, 46, 99, 21, 29, 96, 104, 39, 87, 60, 18, 35, 47, 48, 68, 78, 92, 98, 101, 103, 50, 10, 82, 42, 110, 121, 128 | 28 | 2 | 30 |
| partnerships / links to external stakeholders | 44, 56, 71, 72, 76, 77, 90, 105, 61, 85, 16, 15, 32, 46, 104, 64, 74, 83, 87, 37, 47, 48, 101, 103, 122 - 126 | 24 | 5 | 29 |
| linkages / engagement / alignment with government | 27, 71, 76, 77, 90, 105, 61, 16, 32, 40, 46, 99, 89, 104, 25, 74, 30, 28, 68, 103 | 20 |  | 20 |
| trust | 27, 72, 90, 15, 40, 99, 29, 86, 51, 52, 54, 87, 83, 47, 92, 101, 24 | 17 |  | 17 |
| multidisciplinary | 75, 26, 76, 77, 105, 42, 71, 94, 61, 53, 9, 41, 52, 54, 64, 92 | 16 |  | 16 |
| horizontal / lateral network | 5, 3, 29, 20, 12, 34, 39, 6, 18, 69, 92, 98, 113 – 114, 128 | 13 | 2 | 15 |
| relationships / links between teams / internal | 62, 27, 76, 16, 46, 99, 89, 52, 54, 84, 18, 37, 48, 103, 128 | 15 |  | 15 |
| link community to network | 56, 59, 55, 46, 25, 52, 13, 6, 79, 109, 111 | 9 | 2 | 11 |
| bottom-up / local / organic / informal | 27, 77, 42, 46, 99, 92, 98 | 7 |  | 7 |
| standard / formalized organizational structure | 26, 76, 90, 42, 20, 96, 104 | 7 |  | 7 |
| patient / family / consumer engagement | 33, 71, 72, 90, 64, 101 | 6 |  | 6 |
| vertical structures | 12, 39, 63, 35, 69, 98 | 6 |  | 6 |
| created on existing relationships | 44, 90, 102, 74, 18, 107 | 5 | 1 | 6 |
| voluntary clinician / hospital involvement | 56, 76, 77, 66, 39, 68 | 6 |  | 6 |
| combination of top-down and bottom-up | 56, 72, 61, 39, 113 - 114 | 4 | 2 | 6 |
| mandated / top down on policy and strategic direction | 90, 4, 87, 70 | 4 |  | 4 |
| coordination body | 90, 61 | 2 |  | 2 |
| peer to peer network | 14, 44, 55 | 3 |  | 3 |
| multi-organizational | 75, 61 | 2 |  | 2 |
| open membership | 58, 61 | 2 |  | 2 |
| supra-network | 61 | 1 |  | 1 |

**Table 2:** The component governance and leadership with the corresponding component characteristics and the reference numbers of the selected literature for each characteristic. Number of different published, grey, and total literature sources is included for each characteristic.

| **Governance and Leadership -**Leadership level -Leadership form -Management of network -Governance/decision-making processes | **Publication Number (published + grey literature)** | **# Published** | **# Grey** | **Total #** |
| --- | --- | --- | --- | --- |
| network meetings | 5, 14, 44, 49, 62, 75, 56, 61, 55, 21, 32, 40, 46, 19, 7, 89, 25, 39, 41, 51, 54, 74, 80, 81, 87, 83, 60, 6, 18, 108, 110, 122 – 126, 129 | 30 | 7 | 37 |
| network leadership / management | 27, 56, 71, 72, 76, 77, 42, 85, 43, 24, 15, 53, 40, 99, 104, 9, 25, 52, 54, 63, 74, 81, 87, 95, 35, 48, 78, 79, 92, 98, 50 | 31 |  | 31 |
| working /sub-groups / task forces | 33, 71, 76, 90, 102, 61, 55, 19, 86, 89, 104, 52, 54, 60, 18, 48, 110, 127, 122 - 126 | 16 | 7 | 23 |
| government leadership / oversight | 71, 90, 94, 102, 105, 42, 55, 4, 53, 46, 86, 104, 95, 60, 48, 108, 122 - 126 | 15 | 6 | 21 |
| network manager / coordinator / facilitator | 26, 33, 56, 76, 77, 90, 94, 105, 61, 4, 89, 31, 51, 83, 6, 35, 82, 108 | 17 | 1 | 18 |
| local / clinical champions | 27, 33, 94, 66, 34, 52, 54, 63, 83, 116 - 117, 122 - 126 | 9 | 7 | 16 |
| steering / coordinating committees / groups | 33, 90, 94, 43, 25, 31, 54, 30, 60, 6, 35, 48, 78, 79, 82 | 15 |  | 15 |
| core leadership committee / team | 23, 71, 102, 61, 4, 39, 54, 87, 60, 35, 48, 101, 103 | 13 |  | 13 |
| clinical leader - network coordinator / voluntary co-chairs | 44, 49, 26, 27, 71, 76, 77, 102, 105, 61, 52, 70 | 12 |  | 12 |
| decision-making | 90, 24, 4, 19, 89, 41, 48, 78, 92, 103, 82, 121 | 11 | 1 | 12 |
| interdisciplinary / effective / open / multi governance | 33, 76, 77, 61, 29, 89, 82, 122 - 126 | 7 | 5 | 12 |
| hybrid leadership - clinical (general - specialisms), program/operational, executive | 33, 71, 102, 61, 53, 87, 83, 70, 92, 103 | 10 |  | 10 |
| community / local leadership / ownership in activities | 23, 94, 32, 46, 19, 91, 86, 25, 81 | 9 |  | 9 |
| focal point mid-level managers - clinical leaders | 62, 75, 90, 89, 31, 80, 57, 108 | 7 | 1 | 8 |
| executive support / strategic and technical assistance | 26, 33, 61, 39, 54, 60, 70 | 7 |  | 7 |
| governance structure | 33, 102, 4, 89, 60, 68 | 6 |  | 6 |
| stakeholder management | 44, 33, 71, 4, 41, 82 | 6 |  | 6 |
| administrative core / support | 15, 52, 92 | 3 |  | 3 |

**Table 3:** The component functioning with the corresponding component characteristics and the reference numbers of the selected literature for each characteristic. Number of different published, grey, and total literature sources is included for each characteristic.

| **Functioning** -Management: monitoring compliance, accountability -Clinical: policy/program operationalization, care coordination | **Publication Number (published + grey literature)** | **# Published** | **# Grey** | **Total #** |
| --- | --- | --- | --- | --- |
| knowledge / information sharing / education / learning | 44, 62, 75, 23, 26, 27, 33, 90, 59, 58, 85, 1, 36, 16, 15, 24, 53, 19, 2, 86, 60, 28, 9, 12, 22, 31, 34, 39, 41, 51, 52, 54, 63, 64, 65, 67, 74, 80, 81, 84, 95, 100, 83, 37, 47, 79, 92, 50, 82, 108, 109, 112, 113 - 114, 115, 116 - 117, 118, 119, 127, 122 – 126, 128, 129 | 51 | 16 | 67 |
| guideline / standards / protocols uptake / adherence | 5, 14, 44, 49, 75, 23, 33, 94, 102, 42, 58, 85, 1, 36, 16, 24, 53, 91, 20, 96, 7, 89, 104, 21, 29, 32, 40, 46, 93, 99, 30, 60, 88, 28, 9, 22, 25, 31, 52, 74, 83, 6, 18, 35, 70, 78, 79, 92, 10, 82, 108, 110, 116 - 117, 119, 127, 129 | 51 | 6 | 57 |
| data collection, analysis, use, quality | 5, 14, 49, 62, 75, 23, 33, 42, 55, 16, 24, 91, 86, 89, 21, 32, 40, 46, 93, 99, 60, 97, 9, 22, 39, 41, 51, 52, 54, 63, 64, 65, 67, 74, 80, 84, 95, 100, 48, 78, 79, 92, 50, 108, 109, 110, 111, 116 - 117, 120, 121, 122 - 126 | 43 | 13 | 56 |
| quality improvement | 75, 23, 26, 56, 77, 42, 55, 16, 24, 91, 96, 86, 21, 32, 40, 99, 9, 22, 25, 31, 34, 41, 51, 52, 54, 63, 64, 65, 74, 81, 84, 95, 100, 48, 68, 70, 92, 82, 108, 109, 112, 115, 116 - 117, 121, 122 – 126, 129 | 39 | 12 | 51 |
| care pathways / models of service delivery promotion / implementation | 23, 26, 33, 71, 77, 90, 102, 59, 43, 66, 8, 45, 91, 20, 86, 89, 32, 46, 99, 60, 73, 97, 31, 52, 74, 87, 17, 18, 35, 37, 69, 70, 79, 98, 101, 10, 50, 107, 108, 111, 120, 122 – 126, 128 | 38 | 9 | 47 |
| training and/or supervision | 55, 24, 20, 96, 2, 21, 29, 32, 40, 46, 93, 30, 57, 60, 88, 22, 25, 34, 41, 63, 65, 74, 81, 84, 83, 17, 18, 35, 37, 47, 48, 78, 79, 103, 10, 50, 107, 108, 109, 110, 111, 116 - 117, 119, 127 | 36 | 9 | 45 |
| feedback - performance / performance management / care processes | 5, 14, 44, 49, 62, 75, 94, 102, 42, 4, 8, 91, 21, 29, 13, 60, 9, 63, 65, 100, 17, 68, 70, 78, 79, 92, 82, 116 - 117, 120, 122 - 126 | 27 | 8 | 35 |
| work plans / strategic planning / project development | 27, 33, 56, 72, 76, 77, 91, 32, 40, 30, 60, 28, 64, 87, 100, 83, 47, 68, 103, 121, 122 - 126 | 19 | 6 | 25 |
| revise / standardize patient forms / records systems / tools | 5, 14, 44, 49, 75, 90, 24, 91, 86, 40, 46, 99, 13, 9, 39, 6, 17, 37, 48, 70, 92, 10, 110, 111 | 22 | 2 | 24 |
| mentoring / coaching | 75, 55, 96, 21, 40, 46, 93, 57, 60, 31, 34, 39, 41, 63, 64, 74, 81, 95, 100, 107, 108, 112, 116 - 117 | 19 | 5 | 24 |
| coordination | 27, 90, 94, 1, 36, 8, 45, 91, 29, 30, 73, 47, 48, 69, 92, 98, 107, 122 – 126, 129 | 17 | 6 | 23 |
| changes in practice or service delivery | 76, 94, 102, 15, 53, 104, 99, 13, 60, 9, 25, 41, 65, 67, 47, 48, 108, 116 - 117 | 16 | 3 | 19 |
| monitoring | 14, 16, 96, 86, 21, 29, 99, 30, 17, 18, 35, 121, 122 - 126 | 11 | 6 | 17 |
| reporting | 14, 44, 42, 24, 86, 89, 104, 40, 30, 9, 22, 64, 70, 92, 115, 120 | 14 | 2 | 16 |
| assessment / evaluation | 5, 71, 76, 90, 58, 16, 24, 8, 86, 32, 99, 81, 87, 103 | 14 |  | 14 |
| collaboration | 77, 90, 61, 16, 24, 53, 13, 52, 64, 65, 74, 98, 101, 107 | 13 | 1 | 14 |
| accountability | 72, 42, 91, 89, 60, 52, 65, 47, 68, 78 | 10 |  | 10 |
| performance comparison / benchmarking | 62, 75, 94, 86, 29, 22, 39, 41, 63, 65 | 10 |  | 10 |
| quality of care | 44, 43, 8, 20, 96, 29, 48, 101, 10 | 9 |  | 9 |
| role clarification / orientation of new providers on preferred practices | 62, 72, 46, 51, 87, 92, 98, 111 | 7 | 1 | 8 |
| MPDSR | 21, 32, 40, 46, 60, 108, 120 | 5 | 2 | 7 |
| audit and feedback - data | 44, 49, 27, 53, 84 | 5 |  | 5 |
| leadership training / enhance skills / relationship building | 44, 19, 40, 63 | 4 |  | 4 |
| clinical audit | 75, 7, 40, 82 | 4 |  | 4 |
| teamwork | 105, 54, 92, 98 | 4 |  | 4 |
| shared responsibility | 55, 65 | 2 |  | 2 |
| mentorship and training - management | 14 | 1 |  | 1 |

**Table 4:** The component resources with the corresponding component characteristics and the reference numbers of the selected literature for each characteristic. Number of different published, grey, and total literature sources is included for each characteristic.

| **Resources** -Human resources -Financial | **Publication Number (published + grey literature)** | **# Published** | **# Grey** | **Total #** |
| --- | --- | --- | --- | --- |
| human resources | 14, 62, 27, 33, 76, 90, 94, 105, 61, 36, 15, 53, 29, 32, 46, 93, 99, 12, 39, 100, 83, 57, 73, 28, 48, 70, 79, 92, 101, 10, 50, 20, 89, 40 / 111, 116 - 117, 119, 122 - 126 | 34 | 9 | 43 |
| IT | 14, 27, 33, 76, 58, 55, 66, 16, 53, 21, 32, 40, 46, 8, 31, 54, 64, 73, 18, 35, 69, 70, 98, 101, 38, 104, 92, 36, 81, 48 / 113 - 14, 120, 122 - 126 | 30 | 8 | 38 |
| commodities/equipment | 21, 29, 32, 40, 46, 93, 60, 88, 97, 20, 96, 108, 119 | 12 | 2 | 14 |
| funding | 27, 33, 90, 105, 53, 3, 19, 18, 48, 101, 103, 96, 119 | 12 | 1 | 13 |
| government funding | 26, 71, 59, 61, 21, 60, 35, 48, 68, 78, 103, 38 | 12 |  | 12 |
| supportive policies | 27, 43, 24, 3, 46, 99, 60, 73, 18, 98, 101, 104 | 12 |  | 12 |
| transport | 3, 21, 46, 93, 99, 97, 17, 32, 13, 110, 128 | 10 | 1 | 11 |
| infrastructure | 90, 3, 21, 29, 32, 40, 79, 86, 104 | 9 |  | 9 |
| cost reduction / savings | 43, 66, 45, 91, 67, 48, 70, 101, 47 | 9 |  | 9 |
| financial incentives | 42, 21, 40, 91, 31, 70, 101, 103 | 8 |  | 8 |
| administrative / operational support | 27, 76, 53, 99, 52, 54, 81, 68 | 8 |  | 8 |
| financial subsidies / free services | 36, 21, 32, 17, 37 | 5 |  | 5 |
| UHC / insurance | 21, 99, 31, 60 | 4 |  | 4 |
| pay-for-performance / results | 91, 81, 35, 78 | 4 |  | 4 |
| financial management support | 21, 99, 68, 96 | 4 |  | 4 |
| resource sharing | 19, 69, 82 | 3 |  | 3 |
| service payments | 91, 20 | 2 |  | 2 |
| non-financial incentives | 44, 40 | 2 |  | 2 |

**Table 5:** The component communication with the corresponding component characteristics and the reference numbers of the selected literature for each characteristic. Number of different published, grey, and total literature sources is included for each characteristic.

| **Communication** | **Publication Number (published + grey literature)** | **# Published** | **# Grey** | **Total #** |
| --- | --- | --- | --- | --- |
| between network members | 56, 105, 36, 3, 8, 51, 100, 57, 60, 28, 18, 69, 92, 86, 44, 77, 102, 4, 82, 27, 24, 99, 128, 129 | 24 |  | 24 |
| communication infrastructure | 32, 40, 46, 99, 97, 73, 60, 10, 113 - 114, 120, 129 | 9 | 3 | 12 |
| strengthening communication | 13, 70, 112, 118, 108 | 2 | 3 | 5 |
| effective strategies | 33, 72, 79, 107 | 3 | 1 | 4 |
