# Supplementary material for: Understanding networks in low-and middle-income countries’ health systems: A scoping review
Source: PLOS Glob Public Health. 2023 Jan 11;3(1):e0001387. doi: 10.1371/journal.pgph.0001387 (PMC10022031; doi:10.1371/journal.pgph.0001387)
Supplement: S4 Appendix — (DOCX) [file pgph.0001387.s008.docx]

## S8 Appendix. Reported network uses, purposes, and stakeholders

**Table 1:** Reported network uses with corresponding reference numbers of the selected literature for each reported network use. Number of different published, grey, and total literature sources is included for each reported network use.

| **Use** | **Publication Number (published + grey literature)** | **# Published** | **# Grey** | **Total #** |
| --- | --- | --- | --- | --- |
| provide / improve / expand care / services | 11, 14, 15, 18, 20, 21, 23, 25, 27, 28, 29, 33, 34, 36, 42, 43, 46, 47, 54, 57, 63, 70, 72, 73, 77, 85, 93, 100, 116-117, 128 | 29 | 2 | 31 |
| improve / provide quality and efficiency of care | 8, 21, 22, 25, 29, 32, 33, 42, 46, 60, 67, 93, 98, 102, 107, 108 | 14 | 2 | 16 |
| uptake of standard guidelines / change clinical practice | 5, 6, 9, 31, 34, 39, 44, 53, 54, 61, 62, 64, 67, 75, 94, 118 | 15 | 1 | 16 |
| to change aspects of the health system / service delivery | 19, 23, 27, 69, 72, 76, 77, 84, 87, 101, 102, 103, 109, 112 | 12 | 2 | 14 |
| increase access to care and use | 20, 28, 30, 36, 59, 66, 97, 98, 106, 107, 119, 128 | 10 | 2 | 12 |
| overcome obstacles / gaps to provision of basic services / interventions | 3, 29, 46, 71, 72, 75, 83, 84, 102 | 9 |  | 9 |
| to build capacity / skills | 23, 27, 38, 44, 58, 63, 81, 87, 118 | 8 | 1 | 9 |
| respond to specific health problems | 16, 29, 37, 48, 68, 76, 81, 90, 103 | 9 |  | 9 |
| promote standard / evidence-based approaches to care | 5, 26, 30, 53, 56, 72, 92, 107 | 7 | 1 | 8 |
| foster flow of knowledge and share best practices among providers and organizations and care givers | 4, 38, 58, 59, 71, 74, 115, 118 | 6 | 2 | 8 |
| implementation of QI initiatives | 26, 31, 40, 41, 52, 54, 65, 95 | 8 |  | 8 |
| foster partnerships / teamwork / linkages | 15, 23, 27, 30, 32, 55, 73 | 7 |  | 7 |
| facilitate / improve referral | 17, 38, 50, 60, 88, 106, 128 | 7 |  | 7 |
| to meet people's needs | 36, 47, 48, 90, 98, 102 | 6 |  | 6 |
| manage patients / care | 1, 10, 27, 37, 50, 88 | 6 |  | 6 |
| collaboration | 26, 27, 40, 58, 74, 82 | 6 |  | 6 |
| platform for problem solving | 13, 15, 24, 29, 68, 128 | 6 |  | 6 |
| to facilitate coordination and cooperation | 8, 24, 37, 55, 104, 129 | 6 |  | 6 |
| studies / research / generate evidence | 54, 85, 94, 109, 118 | 3 | 2 | 5 |
| develop / implement / improve models of care / UHC | 29, 51, 55, 61, 103 | 5 |  | 5 |
| coordinate complex care / continuum of care | 29, 32, 42, 47, 93 | 5 |  | 5 |
| introduce new technologies / innovations | 29, 61, 71, 87, 109 | 4 | 1 | 5 |
| shift culture of the network | 6, 39, 44 | 3 |  | 3 |
| use data and evidence to guide decision making | 30, 71, 99 | 3 |  | 3 |
| improve governance | 60, 89, 98 | 3 |  | 3 |
| decentralization of care / distribution of cases | 40, 99 | 2 |  | 2 |
| generate consumer demand | 20, 93 | 2 |  | 2 |
| streamline patient pathways | 4 | 1 |  | 1 |
| consultation | 2 | 1 |  | 1 |
| shift staff attitudes | 6 | 1 |  | 1 |
| deliver care benefits | 7 | 1 |  | 1 |
| improve documentation | 62 | 1 |  | 1 |

**Table 2:** Reported network purposes with corresponding reference numbers of the selected literature for each reported network purpose. Number of different published, grey, and total literature sources is included for each reported network use.

| **Purpose** | **Publication Number (published + grey)** | **# Published** | **# Grey** | **Total #** |
| --- | --- | --- | --- | --- |
| provide optimal / high / improve quality care | 1, 3, 6, 13, 14, 15, 17, 18, 20, 23, 25, 26, 29, 30, 32, 33, 34, 37, 38, 48, 50, 52, 54, 56, 59, 63, 64, 68, 69, 71, 73, 75, 77, 81, 82, 84, 86, 88, 90, 92, 95, 96, 99, 103, 104, 107, 115, 122-126 | 45 | 7 | 52 |
| improve outcomes | 10, 11, 16, 21, 22, 24, 26, 29, 31, 39, 40, 46, 48, 51, 52, 55, 57, 58, 60, 61, 64, 65, 66, 71, 74, 86, 89, 91, 92, 93, 97, 99, 100, 101, 102, 103, 104, 105, 106, 108, 119, 122-126 | 39 | 7 | 46 |
| to transform / improve delivery of services / system | 29, 33, 36, 40, 42, 43, 47, 53, 55, 56, 61, 69, 76, 85, 101, 105, 118 | 16 | 1 | 17 |
| address access to care | 11, 19, 25, 28, 32, 33, 37, 43, 79, 81, 90, 96, 102, 105, 109, 116, 117 | 14 | 3 | 17 |
| to foster practice change / uptake evidence-based practices | 5, 9, 26, 33, 49, 76, 90, 94, 102, 107 | 9 | 1 | 10 |
| improve participation / information / experience to patients / clients | 33, 69, 71, 90, 105, 122-126 | 5 | 5 | 10 |
| address challenges to providing care | 3, 7, 15, 19, 28, 44, 80, 83 | 8 |  | 8 |
| reduce / improve referral | 7, 30, 41, 69, 90, 110 | 5 | 1 | 6 |
| contain / reduce healthcare costs | 10, 28, 96, 101, 104, 115 | 5 | 1 | 6 |
| linking of stakeholders / network entities | 2, 34, 43, 52, 69, 71 | 6 |  | 6 |
| coordinate care | 28, 33, 45, 90 | 4 |  | 4 |
| provide integrated patient / family centered care | 8, 32, 69, 87 | 4 |  | 4 |
| provide information / teaching resources to providers | 7, 33, 85 | 3 |  | 3 |
| reduce hospital admissions / stays | 10, 67, 101 | 3 |  | 3 |
| improve coverage of services | 18, 41, 96 | 3 |  | 3 |
| increase uptake/use of services | 104, 116, 117 | 1 | 2 | 3 |
| reduce incidence of complication | 9, 80 | 2 |  | 2 |
| improve generation and use of health data | 44, 49 | 2 |  | 2 |
| provider feedback | 44 | 1 |  | 1 |

**Table 3:** Reported network stakeholders with corresponding reference numbers of the selected literature for each reported network stakeholder. Number of different published, grey, and total literature sources is included for each reported network use.

| **Stakeholder** | **Publication Number (published + grey literature)** | **# Published** | **# Grey** | **Total #** |
| --- | --- | --- | --- | --- |
| clinicians | 1, 2, 3, 4, 5, 6, 7, 8, 9, 10, 11, 13, 14, 16, 18, 20, 21, 22, 23, 24, 26, 28, 29, 30, 31, 32, 34, 36, 37, 38, 39, 40, 41, 42, 43, 44, 48, 49, 50, 51, 52, 53, 54, 56, 57, 59, 62, 63, 64, 65, 66, 67, 69, 70, 71, 72, 74, 76, 77, 79, 82, 83, 85, 86, 89, 90, 92, 93, 94, 95, 99, 100, 101, 102, 103, 104, 105, 107, 115, 116 - 117, 119, 128, 129 | 79 | 5 | 84 |
| PTFs | 3, 9, 21, 23, 25, 31, 37, 40, 41, 46, 55, 58, 60, 61, 63, 71, 72, 74, 77, 79, 80, 81, 84, 86, 87, 88, 90, 93, 96, 97, 108, 109, 110, 118, 119, 122 - 126 | 30 | 10 | 40 |
| MoH | 5, 25, 32, 35, 37, 44, 47, 49, 62, 63, 68, 75, 81, 82, 84, 93, 102, 106, / 108, 110, 112, 122 - 126 | 18 | 8 | 26 |
| community / local organizations | 15, 19, 25, 56, 59, 60, 63, 66, 71, 72, 73, 79, 81, 83, 87, 88, 101, 105, 106, 107, 113 - 114 | 19 | 3 | 22 |
| patients / clients | 16, 20, 23, 29, 31, 53, 57, 61, 64, 71, 72, 73, 76, 77, 82, 90, 92, 100, 102, 105, 116 - 117 | 20 | 2 | 22 |
| professional associations | 5, 14, 23, 24, 34, 37, 44, 49, 53, 60, 62, 75, 77, 79, 86, 99, 101, 122 - 126 | 17 | 5 | 22 |
| research partners | 5, 14, 15, 16, 23, 24, 30, 34, 39, 44, 49, 61, 64, 71, 72, 75, 77, 85, 92, 102, 105 | 21 |  | 21 |
| CHWs/FCHV / allied health staff | 3, 17, 19, 21, 25, 26, 32, 46, 76, 79, 93, 94, 100, 111, 116 - 117, 120 | 13 | 4 | 17 |
| regional / provincial / state health officials / managers | 13, 15, 18, 31, 40, 41, 42, 46, 47, 56, 60, 71, 86, 93, 95, 106, 107, 129 | 17 | 1 | 18 |
| district health officials | 3, 13, 21, 41, 56, 60, 74, 77, 81, 84, 89, 95, 100, 101, 106, 116 - 117 | 15 | 2 | 17 |
| government | 3, 28, 46, 61, 78, 102, 103, 105, 109, 122 - 126 | 8 | 6 | 14 |
| families / care givers | 3, 16, 17, 21, 23, 28, 29, 64, 71, 72, 92 94, 102 | 13 |  | 13 |
| health authorities / PHC boards / councils | 4, 18, 42, 45, 46, 53, 68, 79, 82, 86, 101, 105, 112 | 12 | 1 | 13 |
| national managers / directorates government | 30, 31, 41, 55, 60, 74, 82, 90, 110, 118 | 8 | 2 | 10 |
| community | 3, 19, 20, 21, 81, 84, 93, 106 | 8 |  | 8 |
| data clerks | 44, 49, 51, 53, 54, 62, 89, 107 | 7 | 1 | 8 |
| network coordinator / manager / chairs | 4, 14, 26, 35, 53, 56, 76, 102 | 8 |  | 8 |
| network management team / committee | 4, 30, 35, 48, 54, 55, 79, 102 | 8 |  | 8 |
| facility administrative staff | 1, 4, 6, 51, 62, 63, 64 | 7 |  | 7 |
| health system leaders / executives / champions | 24, 26, 42, 56, 61, 94, 115 | 6 | 1 | 7 |
| industry / private sector agencies | 16, 60, 78, 96, 105, 113 - 114, | 5 | 2 | 7 |
| hospital service team leader | 14, 39, 65, 76, 89, 99 | 6 |  | 6 |
| policy makers / advisor | 3, 15, 71, 72, 76, 105 | 6 |  | 6 |
| ambulance staff / transport drivers | 1, 46, 82, 93, 106 | 5 |  | 5 |
| local government | 21, 46, 107, 113 - 114 | 2 | 3 | 5 |
| operations leaders / partners | 23, 71, 72, 82, 105 | 5 |  | 5 |
| supervisors / facilitators / mentors | 11, 81, 89, 94, 95 | 5 |  | 5 |
| TBAs | 25, 46, 73, 79, 93 | 5 |  | 5 |
| facility management staff | 39, 43, 81, 89 | 4 |  | 4 |
| health management professionals | 36, 42, 55, 61 | 4 |  | 4 |
| pharmacy workers | 49, 57, 86, 101 | 4 |  | 4 |
| QI personnel | 3, 51, 65, 83 | 4 |  | 4 |
| social workers | 6, 10, 30, 59 | 4 |  | 4 |
| university staff | 2, 7, 14, 70 | 4 |  | 4 |
| counsellors | 41, 57, 59 | 3 |  | 3 |
| hospital associations | 39, 59, 86 | 3 |  | 3 |
| network executive | 76, 102, 104 | 3 |  | 3 |
| other ministries (MoF / MoWCA) | 45, 101, 107 | 2 | 1 | 3 |
| project managers / teams | 54, 83, 94 | 3 |  | 3 |
| social security | 25, 82, 101 | 3 |  | 3 |
| university medical students | 2, 34, 59 | 3 |  | 3 |
| activists / advocacy groups | 55, 59 | 2 |  | 2 |
| health insurers | 82, 99 | 2 |  | 2 |
| IT support | 53, 83 | 2 |  | 2 |
| laboratory staff | 41, 63 | 2 |  | 2 |
| network administrators | 53, 70 | 2 |  | 2 |
| patient associations | 72, 82 | 2 |  | 2 |
| regulatory bodies | 16, 86 | 2 |  | 2 |
| case managers | 19 | 1 |  | 1 |
| decision makers | 23 | 1 |  | 1 |
| elected officials | 19 | 1 |  | 1 |
| facility management committee | 21 | 1 |  | 1 |
| hospital CEO | 39 | 1 |  | 1 |
| legal experts | 72 | 1 |  | 1 |
| school health teams | 6 | 1 |  | 1 |
| service commissioners | 4 | 1 |  | 1 |
| **Levels of System** |  |  |  | **Total #** |
| PHC | 1, 8, 18, 25, 32, 35, 37, 38, 46, 50, 57, 79, 83, 84, 88, 89, 96, 97, 129 | 19 |  | 19 |
| district / county / regional hospital | 4, 5, 25, 32, 49, 59, 60, 62, 75, 88, 89, 94, 128 | 13 |  | 13 |
| private sector | 20, 46, 50, 60, 80, 119, 122 - 126 | 5 | 6 | 11 |
| tertiary / university hospitals | 4, 7, 38, 57, 60, 66, 70, 88, 89, 94, 128 | 10 |  | 10 |
| university | 49, 62, 113 - 114, 122 - 126 | 2 | 7 | 9 |
| hospitals | 63, 80, 84, 87, 108 | 4 | 1 | 5 |
| referral hospital | 11, 17, 37, 84, 97 | 5 |  | 5 |
| medical center | 24 | 1 |  | 1 |
| military health system | 45 | 1 |  | 1 |
| TBA clinics | 46 | 1 |  | 1 |
