# Supplementary material for: Understanding networks in low-and middle-income countries’ health systems: A scoping review
Source: PLOS Glob Public Health. 2023 Jan 11;3(1):e0001387. doi: 10.1371/journal.pgph.0001387 (PMC10022031; doi:10.1371/journal.pgph.0001387)
Supplement: S5 Appendix — (PDF) [file pgph.0001387.s009.pdf]

Networks in health systems: a scoping review

Abstract

Networks exist across the field of healthcare from the global to community level. There is a large and diverse body of research on health-related networks with many types, focuses, uses, and purposes; however, limited literature looks across more than one type of network. A scoping review would be a useful way to organize, map, and summarize the literature on networks in health systems, particularly in low-and middle-income countries (LMICs). Mapping the literature will identify key characteristics, uses, purposes, and definitions of networks as well as show where there are current knowledge gaps, particularly for networks in LMICs. The main objective of this scoping review is to develop an understanding of different types of networks and the purposes for which they are used in health systems through the development of an operational typology of networks in health systems. Literature related to health system networks will be searched in six databases: Medline, EMBASE, Global Health, the Cochrane Library, Web of Science Core Collection, and Global Index Medicus. A data extraction instrument will compile key information and findings from the selected literature relevant to the review question, specifically: intervention country, network definition, stakeholders, type, characteristics, use, and purpose. The data will be presented to highlight networks by intervention country (high-income vs. LMICs), network definitions, stakeholders, type, characteristics, use, and purposes and their frequencies; any other relevant findings will be group by common theme. Drawing from the analysed and presented data, this scoping review will propose an operational typology of networks in health systems.

Introduction

Networks exist across the field of healthcare from global health advocacy networks to expansive service delivery networks. An initial review of the literature found a diverse array of types and definitions of networks, including global networks, inter-organizational networks, communities of practice, clinical networks, quality of care collaboratives, clinical information networks, managed networks, program networks, and networks of care. Networks are developed and implemented for a range of purposes, such as to address variations in practice and outcomes,[i] improve processes and quality of care,[ii] increase evidence-based practice,[iii] facilitate change,[iv] and achieve joint goals.[v] Despite the diversity of networks, it is possible to identify similar characteristics across them, including having a shared communal goal or vision, strong visionary leadership, communication, and trust.[vi,vii,viii,ix] While networks are a phenomenon across many countries, the majority of the published literature on networks is focused in high-incomes countries and therefore, limited research on networks in low-and-middle income countries (LMICs) has been published.

With a large body of research on health-related networks covering many different types, focuses, and purposes, a scoping review would be a useful way to organize, map, and summarize the literature. Limited studies in the literature look across different types of networks and mapping the literature will identify key characteristics, stakeholders, purposes, uses, and definitions of networks as well as show where there are gaps in knowledge, particularly for networks in LMICs. This will lead to the development of an operational typology of networks in health systems.

An initial review of the published literature and subsequent searches have found few scoping or systematic reviews on health system networks. One network related scoping study (Carmone et al. 2020)

Katherine W. Kalaris
01/02/2021

was identified. It employed a scoping study methodology to develop a definition, framework, and evidence base for the service delivery concept, Networks of Care.[x] Two systematic reviews on networks were also identified. Brown et al. (2016) determined the effectiveness of clinical networks and how successful networks improve quality of care and patient outcomes.[xi] Wells et al. (2018) focused on quality improvement collaboratives' improvements on clinical processes and patient outcomes.[xii] A summary of details on these studies, as well as how this study will differ, is provided below. Given the specific nature of these previous studies and the varied literature on networks in health systems identified to be available, a scoping review would be relevant to map and summarize the current literature.

Network	Author	Study	Purpose	Network Definition/Results
Networks of Care	Carmone et al. 2020	Scoping Study<br><br>Quantitative and qualitative studies of evidence and counterfactual<br><br>Stakeholder consultations<br><br>Case studies	• developed a definition and framework and built an evidence base and case studies for the service delivery focused concept, Networks of Care	• "a group of public and/or private health service delivery sites deliberately interconnected through an administrative and clinical management model which promotes a structure and culture that prioritizes client-centered, effective, efficient operation and collaborative learning, enabling providers across all levels of care, not excluding the community, to work in teams and share responsibility for health outcomes"<br>• Development of Networks of Care framework: 1) Agreement and Enabling Environment, 2) Operational Standards, 3) Quality, Efficiency, and Responsibility, 4) Learning and Adaptation<br>• Focused on service delivery networks only, while the evidence base includes network examples, most of the evidence is focused on a specific framework component
Clinical Networks	Brown et al. 2016	Systematic Review<br><br>Quantitative and qualitative studies	• determined the effectiveness of clinical networks and how successful networks improve quality of care and patient outcomes<br>• identified how clinical networks achieved impacts	• "voluntary clinician groupings that aim to improve clinical care and service delivery using a collegial approach to identify and implement a range of quality improvement strategies"<br>• The review found evidence that clinical networks can improve provision of services but there are limited high quality quantitative studies on clinical network effectiveness<br>• Focused on clinical networks only, considered limited evidence
Quality Improvement Collaborative	Wells et al. 2018	Systematic Review<br><br>Cluster RCTs, controlled before-after, interrupter	• evaluated evidence for the impact of quality improvement collaboratives	• "organized, multifaceted approach that includes teams from multiple healthcare sites coming together to learn, apply and share improvement methods, ideas and data on service performance for a given healthcare topic"<br>• Quality improvement collaboratives achieved improvements in targeted clinical processes and patient outcomes, however, less than a third of

		time series studies		studies included met set quality and reporting criteria<br>• Focused on quality improvement collaboratives only, considered limited evidence

Table 1: identified scoping study and systematic reviews on types of networks

The main objective of this scoping review is to develop an understanding of different types of networks, when and what they are used for, and the purposes they intend to achieve through the development of an operational typology of health system networks. Specific objectives of the scoping review are as follows: 1) perform a systematic search of published and grey literature on networks in health systems, with a particular focus on LMICs; 2) map and summarize the type of networks, the network definitions, network stakeholders, network characteristics, network uses, network purposes, and other key finding relevant to networks in health systems; 3) identify gaps in the literature related to networks in LMIC health systems; and 4) propose an operational typology of networks in health systems.

Review question

What is a network, when are they used, what are they used for, and what purposes do they intended to achieve in health systems?

Keywords

Networks, health systems, LMICs, Scoping Review

Methods

This scoping review will be conducted in accordance with the original six phase scoping study framework developed by Arksey and O'Malley (2005) and take into consideration additional recommendations on scoping reviews from Levac et al. (2010) and Peters et al. (2015).[xiii,xiv,xv] The scoping review methodology was selected as it will enable the examination of the extent, range, and nature of the activity of interest, networks in health systems in LMICs, and the identification of gaps in the existing evidence, specifically related to networks in LMICs, from a large body of network related literature.[xvi] The literature on networks in health systems is large and diverse and scoping reviews have been found to be particularly useful when the body of literature is of "large, complex, or heterogeneous nature not amenable to a more precise systematic review."[xvii] Furthermore, the scoping review will help to "clarify a complex concept and refine subsequent research inquiries."[xviii]

Types of sources

This scoping review will consider all types of published and grey literature.

Search strategy

The search strategy has been developed to identify both published and unpublished literature.

1) The first step will consist of searching two databases (PubMed and EMBASE) to identify published literature on networks in health systems in LMICs using an initial list of basic search terms which

describe different types of networks (health system network, clinical network, network of care, inter-organization network, quality improvement collaborative, quality of care collaborative, clinical information networks, managed network, managed clinical network, care network, program network, hybrid network, integrated service delivery network). With literature previously collected and reviewed from high-income countries and LMICs, an analysis of the text words in the title and abstract as well as the index terms will be used to develop the full search strategy.
2) A second more in-depth search with all relevant identified key words and index terms will be performed across the six databases listed below (see Appendix I for example search strategy[xix]).
3) To manage the anticipated volume of literature on networks, the search will initially focus on networks in health systems in LMICs. Following the selection of literature to include, a third search will be performed by combing the reference lists of the included literature for additional relevant references not previously identified; this will be an opportunity to gather literature from high-income countries that is most relevant to networks in LMICs.
4) If this process does not lead to saturation of the concepts or there are gaps in the data extraction template, additional searches on networks in health systems will be performed, not limited to LMICs.
5) Throughout the scoping review, grey literature will also be searched for from a variety of sources, including WHO's database, Global Index Medicus, conferences abstracts, and organization and government project reports and strategic and planning documents as well as documents referenced in the published literature from the World Health Organization, World Bank, and major bilateral organizations.

The search process will be open to literature in all languages but those published in English and French will be given priority for inclusion, as there are no additional resources or capacity to translate from other languages. Literature published between 2000 to 2020 will be eligible for inclusion; an initial review of the published literature did not result in sources with relevant information prior to 2002. The following databases will be searched: Medline, EMBASE, Global Health, the Cochrane Library, and Web of Science Core Collection, as well as Global Index Medicus for publications specific to LMICs and grey literature.

Eligibility criteria

Literature eligible for inclusion in the review will relate to health system networks. Networks refer to groups of facilities and/or healthcare stakeholders (including but not limited to all types of providers, technicians, government officials, professional associations, NGO's, and donors) linked formally or informally, horizontally or vertically, through programs, interventions, activities, or initiatives. Literature will be excluded if the discussed program, intervention, activity, or initiative occurs only in one facility or locality among only one group of actors. Health systems networks will be the focus of the search process, including specific types of networks, such as inter-organizational networks, program networks, managed networks, clinical networks, and different types of service delivery networks. Global level advocacy and coordination networks, cross-country learning networks, and communities of practice will be acknowledged for their roles in global advocacy, policy, and knowledge exchange but will be less of a focus than networks focused on implementation of health programs and clinical services. Research or purely academic networks and networks focused on research capacity building will be excluded as will registry, disaster management, laboratory, diagnostics, social, and home care networks. If other types of networks are identified during the search, they will be included on an ad hoc basis if they align with the outlined eligibility.

As defined by the World Health Organization, a health system "consists of all organizations, people and actions whose primary intent is to promote, restore or maintain health."[xx] Building on this, for the

purposes of eligibility inclusion in this scoping review, health systems will refer to the structures, processes, and people responsible for managing health programs and services that provide care for a population. Literature from both high-income countries and LMICs will be eligible for inclusion in the study, as classified by the World Bank for the 2021 fiscal year.[xxi]

Inclusion	Exclusion
• Relate to health system networks<br>• Networks: groups of facilities and/or healthcare stakeholders (including but not limited to all types of providers, technicians, government officials, professional associations, NGO's, and donors) linked formally or informally, horizontally or vertically, through programs, interventions, activities, or initiatives<br>• Health systems: structures, processes, and people responsible for managing health programs and services that provide care for a population<br>• High-income countries and LMICs	• The program, intervention, activity, or initiative occurs only in one facility or locality among only one group of actors<br>• Research or purely academic networks<br>• Networks focused on research capacity building<br>• Disaster management networks<br>• Database/registry networks<br>• Trial/study networks<br>• Social networks<br>• Family/home care networks<br>• Palliative care networks<br>• Laboratory networks<br>• Diagnostics networks<br>• Disease surveillance networks

Table 2: Inclusion – Exclusion Criteria

Study selection

As each database is searched for all search terms, all citations will be collated and imported into EndNote X9.3.3/2020 (Clarivate Analytics, PA, USA) and duplicates removed. The number of results for each search in each database will be recorded. Citations will be imported into the web application Rayyan,[xxii] a second screening of duplicates will be done, and titles and abstracts will be screened against the inclusion and exclusion criteria. A random sample of at least 10% of the titles and abstracts will be reviewed by a peer to check for consistency in selection. Full text of selected titles and abstracts will be retrieved and assessed against the inclusion and exclusion criteria. A random sample of at least 10% of the full text will be reviewed by a peer to check for consistency in selection. The reasons for exclusion at the full text review will be noted to report. Any discrepancies from the peer screening will be discussed to resolve the conflicts. The results will be reported and presented according to the PRISMA Extension for Scoping Reviews (PRISMA_ScR) guidelines.

Data extraction and charting

Relevant data and insights will be extracted from the included literature by the reviewer with the developed data extraction instrument (Appendix II). This tool will compile key information related to the included studies and findings or insights relevant to the review questions. It will contain the following categories: source citation, intervention country, aim/purpose, methodology, intervention, stakeholders, type of network, network definition, network characteristics, network use, network purpose, and other key finding relevant to the review questions. The data extraction instrument will be piloted with a sample of the literature selected from the full text review and the included grey literature to ensure that it captures all necessary categories of data. If any modifications are made during the piloting, the selected

literature will be rereviewed with the revised instrument. In case the tool is modified during the data extraction and charting process, the changes will be noted and reported. If the modification of the data extraction instrument consists of the addition or changing of the data collection categories, all literature already reviewed for data extraction will be rereviewed to ensure that relevant data is not missed.

Data analysis and Presentation

The following results will be presented from the data extracted and charted:

- Type of network: The different types of networks identified in the selected literature and their frequency will be represented in a chart to show the diversity of networks in health systems
- Network definition: The various network definitions reported in the literature will be presented in a table and their frequency noted where the same definition is used across studies
- Network characteristics: The different characteristics describing networks in the selected literature will be charted by frequency and network to highlight common characteristics across different types of networks; the characteristics will be defined to ensure a common understanding
- Network stakeholders: Types of partners, actors, and stakeholders engaged in the networks described in the selected literature will be charted by frequency and network to show key stakeholders in different networks
- Network use: When and what are the various uses of networks in the selected literature will be charted by frequency and network to show common uses across different types of networks
- Network purpose: The purposes of networks reported in the literature and their frequency will be mapped with their types to show where common networks have similar or dissimilar purposes
- Intervention country: The intervention countries from the selected literature will be grouped by high-income and LMICs and the frequency represented in a chart to show the geographic distribution of research on networks in health systems
- Other key findings: Additional key findings not included in one of the above categories will be grouped by theme and charted according to frequency; this will provide an opportunity to bring light to any notable themes or concepts around networks not anticipated

Drawing on the results described above, a typology of networks in health systems will be proposed.

Consultation

To round out the literature, a consultation with health system experts, particularly those with an interest/experience in networks will be held. This will provide an opportunity to discuss the results and conclusions from the scoping review with an expert audience. This step will be contingent on identification and availability of experts.

Acknowledgements

This scoping review will be the first phase of a doctoral thesis supervised by Dr. Geoff Wong and Prof. Mike English with study selection support from Peter Anto Johnson.

Conflict of interest

There is no conflict of interest.

Appendices

I. Sample Search Strategy for Medline

(health system network* OR clinical network OR networks of care OR inter-organization network* OR managed network* OR care network* OR hybrid network* OR integrated service delivery network* OR managed clinical network* OR program network* OR quality improvement collaborative* OR quality of care collaborative OR healthcare network* OR network based organization* OR network model OR network* relationship* OR networked governance OR integrated healthcare OR chain of care OR clinical community OR practitioner network* OR networks of health services OR delivery of healthcare OR health system planning OR organization of healthcare OR networks of clinical experts OR referral network* OR clinical governance OR quality improvement program* OR network initiative OR local network* OR district governance OR healthcare delivery OR (healthcare organization and administration) OR integrated delivery of healthcare OR network of safety OR collaborative improvement network* OR provincial network of health services OR health network structures OR quality of care network OR regionalized healthcare OR network of healthcare interventions OR collaborative network OR network for healthcare practice improvement OR integrated system of care OR strategic clinical network* OR regional health network* OR integrated care network* OR network for quality improvement).mp.

AND

(health system* OR LMIC* OR developing countr* OR developing nation* OR developing population* OR developing world OR less developed countr* OR less developed nation* OR less developed population* OR less developed world OR lesser developed countr* OR lesser developed nation* OR lesser developed population* OR lesser developed world OR under developed countr* OR under developed nation* OR under developed population* OR under developed world OR underdeveloped countr* OR underdeveloped nation* OR underdeveloped population* OR underdeveloped world OR middle income countr* OR middle income nation* OR middle income population* OR low income countr* OR low income nation* OR low income population* OR lower income countr* OR lower income nation* OR lower income population* OR underserved countr* OR underserved nation* OR underserved population* OR underserved world OR under served countr* OR under served nation* OR under served population* OR under served world OR deprived countr* OR deprived nation* OR deprived population* OR deprived world OR poor countr* OR poor nation* OR poor population* OR poor world OR poorer countr* OR poorer nation* OR poorer population* OR poorer world OR developing econom* OR less developed econom* OR lesser developed econom* OR under developed econom* OR underdeveloped econom* OR middle income econom* OR low income econom* OR lower income econom* OR low gdp OR low gnp OR low gross domestic OR low gross national OR lower gdp OR lower gnp OR lower gross domestic OR lower gross national OR lmic OR lmics OR third world OR lami countr* OR transitional countr* OR Africa OR Asia OR Caribbean OR West Indies OR South America OR Latin America OR Central America OR Afghanistan OR Albania OR Algeria OR Angola OR Antigua OR Barbuda OR Argentina OR Armenia OR Armenian OR Aruba OR Azerbaijan OR Bahrain OR Bangladesh OR Barbados OR Benin OR Byelarus OR Byelorussian OR Belarus OR Belorussian OR Belorussia OR Belize OR Bhutan OR Bolivia OR Bosnia OR Herzegovina OR Hercegovina OR Botswana OR Brasil OR Brazil OR Bulgaria OR Burkina Faso OR Burkina Fasso OR Upper Volta OR Burundi OR Urundi OR Cambodia OR Khmer Republic OR

Kampuchea OR Cameroon OR Cameroons OR Cameron OR Camerons OR Cape Verde OR Central African Republic OR Chad OR Chile OR China OR Colombia OR Comoros OR Comoro Islands OR Comores OR Mayotte OR Congo OR Zaire OR Costa Rica OR Cote d'Ivoire OR Ivory Coast OR Croatia OR Cuba OR Cyprus OR Czechoslovakia OR Czech Republic OR Slovakia OR Slovak Republic OR Djibouti OR French Somaliland OR Dominica OR Dominican Republic OR East Timor OR East Timur OR Timor Leste OR Ecuador OR Egypt OR United Arab Republic OR El Salvador OR Eritrea OR Estonia OR Ethiopia OR Fiji OR Gabon OR Gabonese Republic OR Gambia OR Gaza OR Georgia Republic OR Georgian Republic OR Ghana OR Gold Coast OR Greece OR Grenada OR Guatemala OR Guinea OR Guam OR Guiana OR Guyana OR Haiti OR Honduras OR Hungary OR India OR Maldives OR Indonesia OR Iran OR Iraq OR Isle of Man OR Jamaica OR Jordan OR Kazakhstan OR Kazakh OR Kenya OR Kiribati OR Korea OR Kosovo OR Kyrgyzstan OR Kirghizia OR Kyrgyz Republic OR Kirghiz OR Kirgizstan OR Lao PDR OR Laos OR Latvia OR Lebanon OR Lesotho OR Basutoland OR Liberia OR Libya OR Lithuania OR Macedonia OR Madagascar OR Malagasy Republic OR Malaysia OR Malaya OR Malay OR Sabah OR Sarawak OR Malawi OR Nyasaland OR Mali OR Malta OR Marshall Islands OR Mauritania OR Mauritius OR Agalega Islands OR Mexico OR Micronesia OR Middle East OR Moldova OR Moldovia OR Moldovian OR Mongolia OR Montenegro OR Morocco OR Ifni OR Mozambique OR Myanmar OR Myanma OR Burma OR Namibia OR Nepal OR Netherlands Antilles OR New Caledonia OR Nicaragua OR Niger OR Nigeria OR Northern Mariana Islands OR Oman OR Muscat OR Pakistan OR Palau OR Palestine OR Panama OR Paraguay OR Peru OR Philippines OR Philipines OR Phillipines OR Phillippines OR Poland OR Portugal OR Puerto Rico OR Romania OR Rumania OR Roumania OR Russia OR Russian OR Rwanda OR Ruanda OR Saint Kitts OR St Kitts OR Nevis OR Saint Lucia OR St Lucia OR Saint Vincent OR St Vincent OR Grenadines OR Samoa OR Samoan Islands OR Navigator Island OR Navigator Islands OR Sao Tome OR Saudi Arabia OR Senegal OR Serbia OR Montenegro OR Seychelles OR Sierra Leone OR Slovenia OR Sri Lanka OR Ceylon OR Solomon Islands OR Somalia OR Sudan OR Suriname OR Surinam OR Swaziland OR Syria OR Tajikistan OR Tadzhikistan OR Tadjikistan OR Tadzhik OR Tanzania OR Thailand OR Togo OR Togolese Republic OR Tonga OR Trinidad OR Tobago OR Tunisia OR Turkey OR Turkmenistan OR Turkmen OR Uganda OR Ukraine OR Uruguay OR USSR OR Soviet Union OR Union of Soviet Socialist Republics OR Uzbekistan OR Uzbek OR Vanuatu OR New Hebrides OR Venezuela OR Vietnam OR Viet Nam OR West Bank OR Yemen OR Yugoslavia OR Zambia OR Zimbabwe OR Rhodesia)

Limit to (humans and yr="2000 – 2021")

II. Data extraction instrument

Study #	Citation	Intervention Country	Aim/ purpose of study	Methodology	Intervention	Network Stakeholders	Type of Network	Network Definition	Network Characteristics	Network Use	Network Purpose	Other key findings
Study 1												
Study 2												
Study….												

References

[i] SK, Shah PS, Singhal N, Aziz K, Synnes A, McMillan D, Seshia MM. Association of a quality improvement program with neonatal outcomes in extremely preterm infants: a prospective cohort study. Canadian Medical Association Journal. 2014; 186(13). doi:10.1503 /cmaj.140399.

[ii] McInnes E, Haines M, Dominello A, Kalucy D, Jammali-Blasi A, Middleton S, Klineberg E. What are the reasons for clinical network success? A qualitative study. BMC Health Services Research. 2015; 15:497. doi: 10.1186/s12913-015-1096-5.

[iii] Manns BJ, Wasylak T. Clinical networks: enablers of health system change. CMAJ. 2019; 191:E1299-1305. doi: 10.1503/cmaj.190313.

[iv] Spence K, Henderson-Smart D. Closing the evidence-practice gap for newborn pain using clinical networks. Journal of Paediatrics and Child Health. 2011; 47: 92-98. doi: 10.1111/j.1440-1754.2010.01895.x.

[v] Sheaff R et Schofield J. Chapter 19. Inter-Organizational Networks in Health Care: Program Networks, Care Networks, and Integrated Care. The Oxford Handbook of Health Care Management. Editors Ferlie E, Montgomery K, Reff Pedersen A, 2016. doi: 10.1093/oxfordhb/9780198705109.001.0001.

[vi] Brown BB, Patel C, McInnes E, Mays N, Young J, Haines M. The effectiveness of clinical networks in improving quality of care and patient outcomes: a systematic review of quantitative and qualitative studies. BMC Health Services Research. 2016; 16:360. doi: 10.1186/s12913-016-1615-z.

[vii] Sheaff R et Schofield J. Chapter 19. Inter-Organizational Networks in Health Care: Program Networks, Care Networks, and Integrated Care. The Oxford Handbook of Health Care Management. Editors Ferlie E, Montgomery K, Reff Pedersen A, 2016. doi: 10.1093/oxfordhb/9780198705109.001.0001.

[viii] Wells S, Tamir O, Gray J, Naidoo D, Bekhit M, Goldmann D. Are quality improvement collaboratives effective? A systematic review. BMJ Qual Saf. 2018; 27:226-240. doi: 10.1136/bmjqs-2017-006926.

[ix] Haines, M. M., Brown, B., D'Este, C. A., Yano, E. M., Craig, J. C., Middleton, S., Castaldi, P. A., Pollock, C. A., Needham, K., Watt, W. H., Elliott, E. J., Scott, A., Dominello, A., Klineberg, E., Atkinson, J., Paul, C. & Redman, S. Improving the quality of healthcare: a cross-sectional study of the features of successful clinical networks. Public Health Research & Practice. 2018; 28 (4):1-9. doi:10.17061/phrp28011803.

[x] Carmone AE, Kalaris K, Leydon N, Sirivansanti N, Smith JM, Storey A, Malata A. Developing a Common Understanding of Networks of Care through a Scoping Study. Health Systems and Reform. 2020; 6(2): e1810921. doi: 10.1080/23288604.2020.1810921.

[xi] Brown BB, Patel C, McInnes E, Mays N, Young J, Haines M. The effectiveness of clinical networks in improving quality of care and patient outcomes: a systematic review of quantitative and qualitative studies. BMC Health Services Research. 2016; 16:360. doi: 10.1186/s12913-016-1615-z.

[xii] Wells S, Tamir O, Gray J, Naidoo D, Bekhit M, Goldmann D. Are quality improvement collaboratives effective? A systematic review. BMJ Qual Saf. 2018; 27:226-240. doi: 10.1136/bmjqs-2017-006926.

[xiii] Arksey H and O'Malley L. Scoping studies: toward a methodological framework. International Journal of Social Research Methodology. 2005; 8(1):19-32. doi: 10.1080/1364557032000119616.

[xiv] Levac D, Colquhoun H, O'Brien KK. Scoping studies: advancing the methodology. Implementation Science. 2010; 5(69). doi:10.1186/1748-5908-5-69.

[xv] Peters MDJ, Godfrey CM, Khalil H, McInerney P, Parker D, Soares CB. Guidance for conducting systematic scoping reviews. International Journal of Evidence-Based Healthcare. 2015; 141-146. doi: 10.1097/XEB.0000000000000050.

[xvi] Arksey H and O'Malley L. Scoping studies: toward a methodological framework. International Journal of Social Research Methodology. 2005; 8(1):19-32. doi: 10.1080/1364557032000119616.

[xvii] Peters MDJ, Godfrey CM, Khalil H, McInerney P, Parker D, Soares CB. Guidance for conducting systematic scoping reviews. International Journal of Evidence-Based Healthcare. 2015; 141-146. doi: 10.1097/XEB.0000000000000050.

[xviii] Levac D, Colquhoun H, O'Brien KK. Scoping studies: advancing the methodology. Implementation Science. 2010; 5(69). doi:10.1186/1748-5908-5-69.

[xix] LMIC search string adapted from: Visser ME, Schoonees A, Ezekiel CN, Randall NP, Naude CE. Agricultural and nutritional education interventions for reducing aflatoxin exposure to improve infant and child growth in low- and middle-income countries. Cochrane Database of Systematic Reviews. 2020; 4. doi: 10.1002/14651858.CD013376.pub2.

[xx] World Health Organization. Everybody's Business: Strengthening Health Systems to Improve Health Outcomes-WHO's Framework for Action. 2007. Geneva: World Health Organization.

[xxi] The World Bank. https://datahelpdesk.worldbank.org/knowledgebase/articles/906519-world-bank-country-and-lending-groups.

[xxii] Mourad Ouzzani, Hossam Hammady, Zbys Fedorowicz, and Ahmed Elmagarmid. Rayyan — a web and mobile app for systematic reviews. Systematic Reviews (2016) 5:210, DOI: 10.1186/s13643-016-0384-4.
