# Supplementary material for: Understanding networks in low-and middle-income countries’ health systems: A scoping review
Source: PLOS Glob Public Health. 2023 Jan 11;3(1):e0001387. doi: 10.1371/journal.pgph.0001387 (PMC10022031; doi:10.1371/journal.pgph.0001387)
Supplement: S6 Appendix — (DOCX) [file pgph.0001387.s010.docx]

Literature identified through database searches

N = 11,142

Medline: 2662 Global Health: 1678

Embase: 4325 Web of Science: 1636

Cochrane: 248 Global Index Medicus (AIM): 593

Identification

#

Additional literature identified

N_p_= 32*

N_g_= 23

Literature after duplicates removed

N = 7,111

Literature excluded

N = 6,818

Duplicates excluded N = 13

Literature excluded during full text retrieval

N = 46

Screening

Literature screened

N = 7,111

Literature excluded

N_p_= 128

N_g_= 32

Conference abstracts N = 3***

Clinical trial registration N = 1

Protocol N = 7

Language N = 3

Unable to find citation/text N = 1

Insufficient information on the network N = 36

Irrelevant N = 105

Theory focused N = 4

Full text literature assessed for eligibility

N_p_= 234

N_g_= 53**

Eligibility

Full text literature included in synthesis

N_p_= 106

N_g_= 21

Included

Update search

Identified N= 2073

Screened N= 1080

Full text assessed N=22

Included N=2

Included

N_p_= 108

N_g_= 21

N_p_ = published literature

N_g_ = grey literature

*Includes 24 previously identified pieces of literature, four identified during the grey literature search, and four from abstracts and clinical trial registrations retrieved from the database searches

**Includes 30 pieces of grey literature that were identified during the screening of the results from the database searches

***Three conference abstracts identified during the full text review were moved to the grey literature review and therefore excluded twice
