# Supplementary material for: Understanding networks in low-and middle-income countries’ health systems: A scoping review
Source: PLOS Glob Public Health. 2023 Jan 11;3(1):e0001387. doi: 10.1371/journal.pgph.0001387 (PMC10022031; doi:10.1371/journal.pgph.0001387)
Supplement: Attachment — Submitted filename: response to reviewers.docx [file pgph.0001387.s012.docx]

15^th^ November 2022

Dear Dr. Barman,

Thank you for providing the opportunity to submit a revised draft of our manuscript **Understanding networks in low-and middle-income countries’ health systems: a scoping review** to *PLOS Global Public Health*. We appreciate the time and effort that your team and the reviewers have dedicated to providing valuable feedback on our manuscript. In the revised version, we have incorporated changes to reflect the suggestions provided by the editors and reviewers. Please find in the table below a point-by-point response to the comments.

We hope this revised manuscript sufficiently address the feedback. Thank you again for considering our manuscript in PLOS Global Public Health.

Sincerely,

Katherine Kalaris

Corresponding author

| **Comments** | **Response** |
| --- | --- |
| Methods: Methods are clear and rigorous. One clarification that can perhaps help, is to include why you chose the databases you chose (for example is it to ensure you cover diversity of fields or to include all health system related papers). The flow chart has a step of identifying databases - so just expanding a bit on why the databases were chosen using two to three sentences will help. | We have added details on the selection of databases, please see Pages 7-8, lines 178-183 |
| Results: The phrases under each main sub-heading such as Improving and providing quality care services seems unclear. Why were those phrases picked? Were they keywords in papers? there are general terms such as 'care' that needs some explaining. Adding a bit more explanations on what Table 4 and 5 represent, leading up to the table, beyond identifying the main sub-headings will bring clarity. | We have added details in the results section on the labelling of the components and categories, and clarified Tables 4 and 5, please see Page 12, lines 289-290 and Pages 19-20, lines 400-418 |
| Limitations: One optional suggestion is to perhaps to mention that not all networks (even though health related) maybe captured in academic literature - therefore may not be included in this review. Mainly because many health networks in LMICs at lower level admin levels do not necessarily get represented in academic literature. | Thank you for the suggestion, this has been included in Pages 28, lines 590-593 |
